# Autoimmune Sequelae After Delta or Omicron Variant SARS-CoV-2 Infection in a Highly Vaccinated Cohort

**DOI:** 10.1001/jamanetworkopen.2024.30983

**Published:** 2024-08-30

**Authors:** Liang En Wee, Jue Tao Lim, An Ting Tay, Calvin J. Chiew, Benjamin Ong, David Chien Boon Lye, Manjari Lahiri, Kelvin Bryan Tan

**Affiliations:** 1 National Centre for Infectious Diseases, Singapore; 2 Duke-NUS Graduate Medical School, National University of Singapore, Singapore; 3 Department of Infectious Diseases, Singapore General Hospital, Singapore; 4 Lee Kong Chian School of Medicine, Nanyang Technological University, Singapore; 5 Ministry of Health, Singapore; 6 Saw Swee Hock School of Public Health, National University of Singapore, Singapore; 7 Department of Medicine, Yong Loo Lin School of Medicine, National University of Singapore, Singapore; 8 Department of Infectious Diseases, Tan Tock Seng Hospital, Singapore; 9 Division of Rheumatology, Department of Medicine, National University Hospital, Singapore

## Abstract

**Importance:**

Studies have reported increased risk of autoimmune sequelae after SARS-CoV-2 infection. However, risk may potentially be attenuated by milder Omicron (B.1.1.529) variant infection and availability of booster vaccination.

**Objective:**

To estimate the 300-day risk of new-incident autoimmune sequelae after SARS-CoV-2 Delta or Omicron BA.1 or BA.2 variant infection in adults who received COVID-19 vaccines and boosters, compared with a contemporary control group without infection.

**Design, Setting, and Participants:**

This cohort study in Singapore enrolled adults from September 1, 2021, to March 7, 2022, and followed up for 300 days. Participants were adults aged 18 years or older with SARS-CoV-2 infection during the predominance of the Delta and Omicron BA.1 or BA.2 variants and were still alive at 30 days after COVID-19 diagnosis.

**Exposure:**

The national SARS-CoV-2 testing registry was used to construct cohorts of adults with SARS-CoV-2 Delta or Omicron BA.1 or BA.2 variant infection (hereafter, cases) and a contemporaneous group with negative polymerase chain reaction or rapid antigen test results (hereafter, controls).

**Main Outcomes and Measures:**

New-incident autoimmune diagnoses after SARS-CoV-2 infection. This information was recorded in the MediClaims national health care claims database and identified 31 to 300 days after index date of infection. Risks and excess burdens were estimated using Cox proportional hazards regression model with overlap weights applied.

**Results:**

In total, 1 766 036 adults (915 096 females [51.9%]; mean [SD] age, 49 [18] years) were included in the study population, with 480 082 (27.2%) categorized as cases and 1 285 954 (72.8%) as controls. Of these adults, 73.1% had Chinese, 13.7% Malay, and 9.9% Indian ethnicity. There were 104 179 cases and 666 575 controls included during the Delta variant–predominance transmission, while 375 903 cases and 619 379 controls were included during the Omicron variant–predominance transmission. During the Delta variant period, 81.1% of cases had completed primary vaccination; during the Omicron variant period, 74.6% of cases received boosters. No significantly elevated risk of 12 prespecified autoimmune sequelae was recorded across the Omicron and Delta variant cohorts. Elevated risks of inflammatory bowel disease (adjusted hazard ratio [AHR], 2.23; 95% CI, 1.45-3.46; *P* < .001) and bullous skin disorders (AHR, 4.88; 95% CI, 2.47-9.66; *P* < .001) were observed only in the subset of COVID-19 cases requiring hospitalization during the predominance of the Omicron variant. While elevated risk of vasculitis (AHR, 5.74; 95% CI, 1.48-22.23; *P* = .01) was observed in vaccine-breakthrough Omicron variant infections, no increased risk of vasculitis was observed in the corresponding subgroup who received boosters.

**Conclusions and Relevance:**

This cohort study observed no significantly elevated long-term risk of autoimmune sequelae after SARS-CoV-2 Delta and Omicron BA.1 or BA.2 variant infection, except for a modestly increased risk of inflammatory bowel disease and bullous skin disorders in the hospitalized subgroup during the predominance of the Omicron variant. Booster vaccination appeared to mitigate the risk of long-term autoimmune sequelae.

## Key Points

**Question:**

What is the long-term risk of autoimmune diagnoses after infection from SARS-CoV-2 Delta or Omicron variant infection in vaccinated cohorts who also received boosters against COVID-19?

**Findings:**

In this cohort study of 1 766 036 adults in Singapore, no significantly elevated risk of autoimmune sequelae after infection from Delta and Omicron BA.1 or BA.2 variants was observed. A modest increase in risk of inflammatory bowel disease and bullous skin disorders was found in the hospitalized subgroup during the predominance of the Omicron variant.

**Meaning:**

Findings of this study suggest that booster vaccination may mitigate the risk of long-term autoimmune sequelae after Omicron variant infection.

## Teaser

This cohort study examines the risk of developing an autoimmune condition after a SARS-CoV-2 Delta or Omicron variant infection among adults in Singapore, which has high COVID-19 testing, vaccination, and booster rates.

## Introduction

Autoimmunity has been reported in patients with severe COVID-19,^1,2^ with development of autoantibodies associated with risk of postacute sequelae.^2^ Increased rates of autoimmune sequelae after SARS-CoV-2 infection have been reported from multiple large-scale population-based electronic health record (EHR) studies.^3,4,5,6,7,8,9,10^ However, several of these studies were conducted during the initial pandemic wave with ancestral SARS-CoV-2 variants and predated populationwide rollout of vaccination.^3,4,5^ Autoantibody prevalence after COVID-19 is associated with greater severity of initial infection.^11,12^ COVID-19 vaccination, predominantly with messenger RNA (mRNA) vaccines, has demonstrated some degree of protection against postacute sequelae across multiple organ systems.^13^ During the current era of COVID-19 endemicity dominated by milder Omicron (B.1.1.529) variants and availability of COVID-19 booster vaccines, reduced severity of acute illness attributed to milder infection and booster vaccination may potentially translate to lower long-term risk of postacute autoimmune sequelae.

However, while increased risk of autoimmune diseases after SARS-CoV-2 Omicron variant infection has been observed across several retrospective population-based cohort studies,^6,7,8^ clinical evidence of the potential protection from vaccination has been mixed. In a large EHR study extending into the predominance of the Omicron variant and including almost 4 million patients, risk of autoimmune disease after infection was higher in the vaccinated group than the unvaccinated group, although fewer than 10% of the patients were vaccinated.^6^ In contrast, vaccination was associated with reduced risk of autoimmune disease after COVID-19 in other population-based cohort studies from the pre-Omicron and Omicron variant periods,^7,9,10^ although the risk of autoimmune sequelae was not specifically evaluated in cohorts who received boosters. In this study, we aimed to estimate the 300-day risk of new-incident autoimmune sequelae after SARS-CoV-2 Delta (B.1.617.2) and Omicron BA.1 or BA.2 variant infection in adults who received COVID-19 vaccines and boosters compared with a contemporary control group without infection.

## Methods

This cohort study was part of national public health research under the Infectious Diseases Act of Singapore. In accordance with the Infectious Diseases Act, individual patient consent and separate ethics review by an institutional review board were not required. We followed the Strengthening the Reporting of Observational Studies in Epidemiology (STROBE) reporting guideline.

### Study Setting and Databases

Singapore is a multiethnic city-state in Asia with a population of 5.4 million. The national SARS-CoV-2 testing registry was used to construct cohorts of adult Singaporeans (ie, citizens or permanent residents of Singapore) with first SARS-CoV-2 infection during periods of community transmission from September 1, 2021, to March 7, 2022, predominated by the Delta and Omicron BA.1 or BA.2 variants. The Delta variant predominated community transmission by September 2021; in December 2021, the Omicron BA.1 or BA.2 variant displaced the Delta variant as the predominant strain.^14^ SARS-CoV-2 infection was defined as a positive result on either a polymerase chain reaction (PCR) test or rapid antigen test (RAT) recorded in the national registry. During the study period, all Singaporeans with acute respiratory illness were strongly encouraged to seek free confirmatory testing for SARS-CoV-2 infection at a health care facility or practitioner. Testing was mandatory for individuals presenting with acute respiratory illness symptoms, and all positive results were reported to the local Ministry of Health.^12^ Outcomes were compared across vaccination status and severity of initial infection (ambulatory care vs hospitalization) using national databases of COVID-19 hospitalizations and vaccinations. Under the national vaccination program, BNT162b2 (Pfizer/BioNTech) and mRNA-1273 (Moderna) were originally approved for use in a 2-dose primary mRNA vaccination series, with subsequent recommendation of boosters.^15^ By March 2022, 91% of the population had completed a primary vaccination series and 69% had received booster doses,^15^ with 95% or more receiving mRNA vaccines.

Risk of prespecified new-incident autoimmune sequelae was assessed using the national health care claims database (MediClaims), encompassing all public and private inpatient and outpatient health care practices. Participation in the national government–administered medical savings scheme (MediSave) and national health insurance plan (MediShield) is mandatory for all Singaporeans.^16^ This environment enabled comprehensive data capture across different health care settings.

#### Cohort

A flowchart of cohort construction is provided in the Figure. Individuals who were aged 18 years or older and were citizens or permanent residents of Singapore were enrolled. The cases group (hereafter, cases) consisted of individuals with SARS-CoV-2 Delta and Omicron BA.1 or BA.2 variant infection. A contemporaneous control group (hereafter, controls), composed of individuals with a documented negative PCR test or RAT result, was drawn from the same population presenting to health care settings for acute respiratory illness symptoms (Delta variant: September 1 to November 30, 2021; Omicron variant: December 1, 2021, to March 7, 2022).

**Figure.**
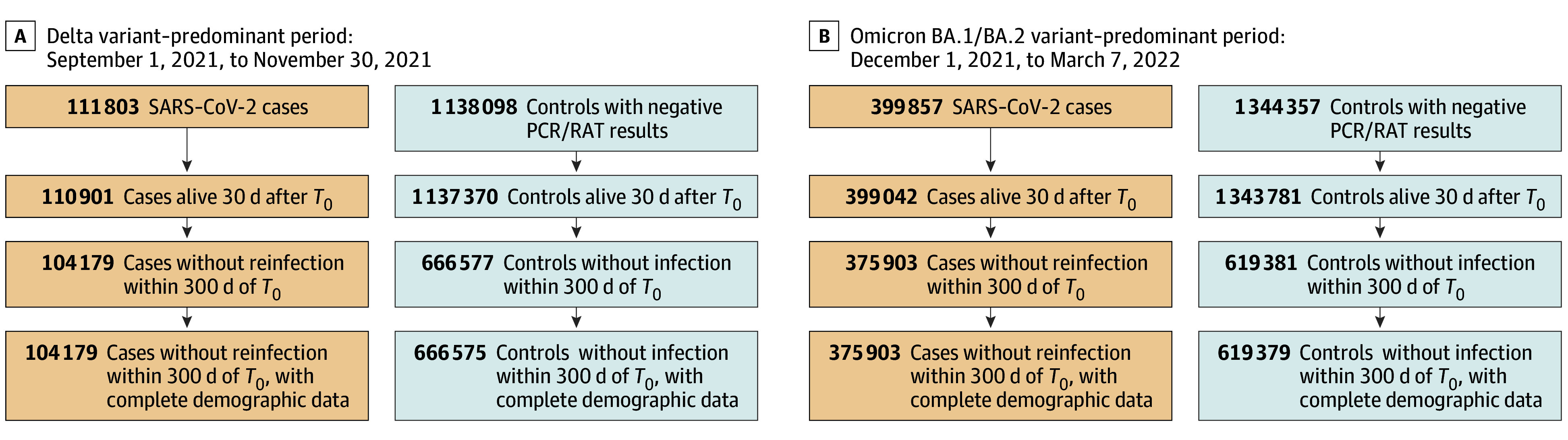
Flowchart of Cohort Construction PCR indicates polymerase chain reaction; RAT, rapid antigen test; T_0_, the date of the first positive PCR or RAT test result in cases and the date of the negative test results in controls.

Individuals who died within the first 30 days of the index date (date of first positive PCR or RAT result in cases; date of negative test result in controls) or with missing sociodemographic data were excluded. Individuals with documented reinfections within 300 days of the index date were also excluded.

#### Prespecified Autoimmune Sequelae

New-incident autoimmune sequelae were assessed during a follow-up period beginning more than 30 days after the index date and ending 300 days after the index date. Diagnoses used *International Statistical Classification of Diseases and Related Health Problems, Tenth Revision* (*ICD-10*) codes as recorded in the national health care claims database.^3,4,5,6,7,8,9,10^ The following prespecified autoimmune sequelae included connective tissue diseases (systemic lupus erythematosus; Sjögren syndrome; systemic sclerosis; dermatomyositis or polymyositis; and other connective tissue diseases, including mixed connective tissue disease), vasculitis (polyarteritis nodosa, antineutrophilic cytoplasmic antibody–associated vasculitis, Takayasu arteritis, and giant cell arteritis), inflammatory arthritis (rheumatoid arthritis and spondyloarthropathies), inflammatory bowel disease, psoriasis, bullous skin disorders (pemphigus and pemphigoid), and autoimmune thyroiditis.^3,4,5,6,7,8,9,10^ The *ICD-10* codes used for outcomes of interest are listed in the eAppendix in Supplement 1.

#### Covariates

The following covariates were incorporated: demographics (age, sex, and ethnicity), vaccination status at the index date, comorbidity burden (Charlson Comorbidity Index [range: 0 to ≥5, with higher scores indicating more severe burden]), and socioeconomic status (SES). Information on sociodemographic characteristics (including ethnicity) was defined based on information recorded in national databases maintained by the Ministry of Health. Ethnicity was assessed in this study to evaluate if risks of autoimmune sequelae differed across ethnic groups. Housing type was used as a surrogate marker of SES.^17^ The majority of Singaporeans (≥90%) live in owner-occupied public housing under a tiered subsidy scheme, with the purchase eligibility for more highly subsidized smaller-sized flats dependent on monthly household income.^17^

### Statistical Analysis

Risks and excess burdens of prespecified new-incident autoimmune sequelae after SARS-CoV-2 infection were estimated using controls as a comparator at 31 to 300 days from the index date. For estimation of risks for each new-incident sequelae, a subcohort of individuals without a history of the studied complication being reported was constructed. Individuals were excluded from each subcohort if they had a preexisting history of the specific sequelae being studied in the past 5 years.

Baseline sociodemographic characteristics of cases with Delta or Omicron variant infection and controls were described, along with standardized mean differences (SMDs) between groups, at baseline and after adjustment using overlap weighting^18^ and incorporating all available covariates: demographic characteristics (age, sex, and ethnicity), SES (housing type), COVID-19 vaccination status at the index date (not fully vaccinated, fully vaccinated, or received boosters), and comorbidities. An SMD less than 0.1 was taken as the threshold for good covariate balance after weighting. Hazard ratios (HRs) of new-incident autoimmune sequelae between cases and controls were then estimated using Cox proportional hazards regression models, with overlap weights applied. Excess burden per 1000 persons at 300 days of follow-up was defined as the excess number of a specific new-incident autoimmune sequelae due to COVID-19 per 1000 persons in the 300-day follow-up period. Excess burden was computed based on the differences in weighted incidence rates of the prespecified postacute sequelae between cases and controls over the 300-day follow-up period. Risks and excess burden of new-incident autoimmune sequelae after SARS-CoV-2 infection were also stratified by vaccination status (fully vaccinated vs received boosters) and severity of initial infection (ambulatory care vs hospitalization). Subgroup analyses by age (18-64 years vs ≥65 years), ethnicity (Chinese, Malay, or Indian), and sex (male vs female) were also conducted.

Additional analyses were performed to investigate the robustness of the results. First, the risk of a series of negative outcomes (various malignant neoplasms) was evaluated across the entire study cohort. These outcomes were chosen because no prior knowledge, to date, supports the existence of a causal association between SARS-CoV-2-infection and cancer risk, with increased cancer risk not reported in survivors of severe acute respiratory syndrome coronavirus and Middle East respiratory syndrome coronavirus.^19^ Second, use of inverse probability weighting (computed as 1/propensity score for cases; 1/[1 – propensity score] for controls) as an alternative to overlap weighting in the regression model was explored. Third, although we focused on prespecified hypotheses and end points, rather than every possible comparison, results with *P* < .004 were considered additionally robust to correction for multiple comparisons (Bonferroni correction).

A 95% CI that excluded 1 was considered to be statistically significant. Analyses were conducted using Stata, version 16 (StataCorp LLC).

## Results

In total, 1 766 036 adult Singaporeans (850 940 males [48.2%], 915 096 females [51.9%]; mean [SD] age, 49 [18] years) were included in the study population, with 480 082 (27.2%) categorized as cases and 1 285 954 (72.8%) as controls. Of these adults, 73.1% had Chinese, 13.7% Malay, and 9.9% Indian ethnicity. There were 104 179 cases with new-onset infection during the Delta variant–predominant period compared with 666 575 controls. Meanwhile, 375 903 cases had new-onset infection during the Omicron BA.1 or BA.2 variant period compared with 619 379 controls (Figure).

As data were obtained from comprehensive national databases, missing data were minimal, and there was no loss to follow-up. Baseline sociodemographic and clinical characteristics are presented in Table 1. During the Delta variant period, 81.1% of cases had completed a primary vaccination series only and 10.6% received boosters. During the Omicron variant period, 22.2% of cases had completed a primary vaccination series only and 74.6% received boosters. During the predominance of the Delta variant, 9.9% of cases were hospitalized, with 1.9% progressing to severe COVID-19. During the predominance of the Omicron variant, 2.5% of cases were hospitalized, with 0.4% progressing to severe COVID-19. After overlap weighting, differences in sociodemographic and clinical characteristics were minimal (all SMDs <0.10) (Table 1).

**Table 1.** Baseline Sociodemographic and Clinical Characteristics of Study Population With Standardized Mean Differences Before and After Overlap Weighting

Sociodemographic and clinical characteristics	Delta variant				Omicron variant			
	No. (%)		SMD^a^		No. (%)		SMD^a^	
	SARS-CoV-2 cases (n = 104 179)	Controls (n = 666 575)	Baseline	Weighted	SARS-CoV-2 cases (n = 375 903)	Controls (n = 619 379)	Baseline	Weighted
Age, mean (SD), years	51 (17)	49 (17)	0.12	0.00	48 (17.66)	47 (17.35)	0.03	0.00
Vaccination status^b^								
Did not complete primary vaccination series	8602 (8.3)	53 965 (8.1)	0.01	0.00	12 143 (3.2)	18 258 (3.0)	0.02	0.00
Completed primary vaccination series only	84 518 (81.1)	519 386 (77.9)	0.08	0.00	83 506 (22.2)	209 415 (33.8)	0.26	0.00
Received ≥1 booster vaccination doses	11 059 (10.6)	93 224 (14.0)	0.10	0.00	280 254 (74.6)	391 706 (63.2)	0.25	0.00
Ethnicity								
Chinese	71 452 (68.6)	491 727 (73.8)	0.11	0.00	272 943 (72.6)	455 466 (73.5)	0.02	0.00
Indian	11 671 (11.2)	67 706 (10.2)	0.03	0.00	30 412 (8.1)	64 339 (10.4)	0.08	0.00
Malay	18 320 (17.6)	84 883 (12.7)	0.14	0.00	62 131 (16.5)	77 459 (12.5)	0.11	0.00
Other ethnicity^c^	2736 (2.6)	22 259 (3.3)	0.04	0.00	10 417 (2.8)	22 115 (3.6)	0.05	0.00
Sex								
Male	58 836 (56.5)	313 750 (47.1)	0.19	0.00	184 483 (49.1)	293 871 (47.5)	0.03	0.00
Female	45 343 (43.5)	352 825 (52.9)	0.19	0.00	191 060 (50.8)	325 508 (52.5)	0.03	0.00
Housing type								
Public housing								
1-2 Rooms	8667 (8.3)	32 422 (4.9)	0.14	0.00	21 530 (5.7)	29 569 (4.8)	0.04	0.00
3 Rooms	23 496 (22.6)	107 830 (16.2)	0.16	0.00	68 436 (18.2)	98 560 (15.9)	0.06	0.00
4 Rooms	36 253 (34.8)	214 040 (32.1)	0.06	0.00	129 822 (34.5)	194 400 (31.4)	0.07	0.00
5 Rooms	23 895 (22.9)	162 886 (24.4)	0.04	0.00	96 071 (25.6)	149 599 (24.2)	0.03	0.00
Private housing	10 526 (10.1)	146 254 (21.9)	0.33	0.00	57 336 (15.3)	143 330 (23.1)	0.20	0.00
Others	1342 (1.3)	3143 (0.5)	0.09	0.00	2708 (0.7)	3921 (0.6)	0.01	0.00
Comorbidity burden (CCI)^d^								
None (CCI = 0)	82 880 (79.6)	563 709 (84.6)	0.13	0.00	306 983 (81.7)	528 978 (85.4)	0.10	0.00
Mild (CCI = 1-2)	14 825 (14.2)	77 261 (11.6)	0.08	0.00	48 329 (12.9)	68 022 (11.0)	0.06	0.00
Moderate (CCI = 3-4)	4290 (4.1)	17 791 (2.7)	0.08	0.00	13 506 (3.6)	15 531 (2.5)	0.06	0.00
Severe (CCI = ≥5)	2184 (2.1)	7814 (1.2)	0.07	0.00	7085 (1.9)	6848 (1.1)	0.06	0.00

Abbreviations: CCI, Charlson Comorbidity Index; mRNA, messenger RNA; SMD, standardized mean difference.

^a^ The SMDs at baseline and after overlap weighting of cases (individuals with SARS-CoV-2 infection) and controls (individuals with negative polymerase chain reaction/rapid antigen test results) were weighted from original samples. An SMD less than 0.1 was the threshold for good covariate balance after weighting.

^b^ Did not complete primary vaccination series was defined as either unvaccinated or partially vaccinated with a single dose of an mRNA COVID-19 vaccine (either BNT162b2 or mRNA-1273). Completed primary vaccination series only was defined as receiving 2 doses of an mRNA COVID-19 vaccine at least 8 weeks apart. Received 1 or more booster vaccination doses was defined as receiving at least a third dose of an mRNA COVID-19 vaccine 6 to 9 months after the second dose.

^c^ Includes other ethnicities (eg, Eurasian and Arab) or multiple ethnicities.

^d^ Comorbidity burden was defined using the CCI (range: 0 to ≥5, with higher scores indicating more severe burden), which consists of the following comorbidities: myocardial infarction, chronic heart failure, peripheral vascular disease, cerebrovascular accident, dementia, chronic obstructive pulmonary disease, connective tissue disease, peptic ulcer disease, diabetes, hemiplegia, liver disease, moderate to severe kidney impairment, solid tumor, leukemia, and HIV infection with AIDS.

There was no significantly increased risk of new-incident autoimmune sequelae observed after SARS-CoV-2 Delta or Omicron variant infection compared with controls (Table 2). Increased risk of Sjögren syndrome (adjusted HR [AHR], 1.91; 95% CI, 1.01-3.63; *P* = .04) and bullous skin disorders (AHR, 1.57; 95% CI, 1.06-2.33; *P* = .03) was observed after Omicron variant infection compared with controls; however, results did not cross the threshold of significance when adjusted for multiple comparisons (Table 2). When stratified by initial infection severity, significantly elevated risks of inflammatory bowel disease (AHR, 2.23; 95% CI, 1.45-3.46; *P* < .001) and bullous skin disorders (AHR, 4.88; 95% CI, 2.47-9.66; *P* < .001) were observed only in the subset of cases requiring hospitalization during the predominance of the Omicron variant, contrasted against controls (Table 3; eTables 1-2 in Supplement 1). While elevated risk of vasculitis (AHR, 5.74; 95% CI, 1.48-22.23; *P* = .01) was observed in vaccine-breakthrough Omicron variant infections compared with controls, no increased risk of vasculitis (AHR, 0.96; 95% CI, 0.49-1.88; *P* = .90) was observed in the corresponding subgroup who received boosters (Table 4; eTables 3-4 in Supplement 1).

**Table 2.** Risks and Excess Burdens of Prespecified New-Incident Autoimmune Diagnoses in SARS-CoV-2 Cases and Controls During Delta and Omicron BA.1 or BA.2 Variant Periods

Autoimmune diagnosis	Cases with autoimmune diagnosis, No. (%)^a^	Cases, No.^a^	Controls with autoimmune diagnosis, No. (%)^a^	Controls, No.^a^	Excess burden, weighted, per 1000 persons (95% CI)^b^	AHR (95% CI)^c^^,^^d^	*P* value
**Delta variant period**							
Systemic lupus erythematosus	2 (<0.01)	104 125	47 (0.01)	666 367	−0.04 (−0.10 to 0.02)	0.31 (0.08 to 1.30)	.11
Rheumatoid arthritis	34 (0.03)	103 902	250 (0.04)	665 098	−0.06 (−0.24 to 0.11)	0.83 (0.58 to 1.20)	.33
Sjögren syndrome	1 (<0.01)	104 162	11 (<0.01)	666 463	−0.01 (−0.04 to 0.03)	0.54 (0.07 to 4.25)	.56
Systemic sclerosis	1 (<0.01)	104 170	6 (<0.01)	666 535	NA^e^	NA^e^	NA^e^
Dermatomyositis or polymyositis	0	104 168	28 (<0.01)	666 515	NA^e^	NA^e^	NA^e^
Other connective tissue diseases^f^	2 (<0.01)	104 169	8 (<0.01)	666 531	0.01 (−0.03 to 0.05)	1.90 (0.40 to 9.13)	.42
Vasculitis	2 (<0.01)	104 144	37 (0.01)	666 363	−0.04 (−0.10 to 0.02)	0.32 (0.08 to 1.33)	.12
Inflammatory bowel disease	66 (0.06)	103 806	422 (0.06)	664 169	−0.03 (−0.27 to 0.21)	0.95 (0.73 to 1.24)	.71
Spondyloarthropathies	10 (0.01)	104 125	64 (0.01)	666 265	−0.02 (−0.11 to 0.07)	0.83 (0.42 to 1.63)	.59
Psoriasis	31 (0.03)	103 883	210 (0.03)	665 114	−0.07 (−0.24 to 0.10)	0.81 (0.56 to 1.19)	.29
Bullous skin disorders	11 (0.01)	104 139	62 (0.01)	666 401	−0.03 (−0.12 to 0.07)	0.79 (0.42 to 1.51)	.48
Autoimmune thyroid disease	20 (0.02)	104 050	142 (0.02)	665 825	−0.02 (−0.16 to 0.11)	0.89 (0.55 to 1.43)	.63
**Omicron BA.1 or BA.2 variant period**							
Systemic lupus erythematosus	31 (0.01)	375 722	58 (0.01)	619 189	−0.01 (−0.06 to 0.05)	0.91 (0.58 to 1.41)	.66
Rheumatoid arthritis	151 (0.04)	374 920	221 (0.04)	618 087	0.05 (−0.06 to 0.17)	1.15 (0.93 to 1.41)	.20
Sjögren syndrome	21 (0.01)	375 833	18 (<0.01)	619 290	0.03 (−0.01 to 0.06)	1.91 (1.01 to 3.63)	.04
Systemic sclerosis	10 (<0.01)	375 873	12 (<0.01)	619 348	0.01 (−0.02 to 0.03)	1.25 (0.53 to 2.90)	.61
Dermatomyositis or polymyositis	6 (<0.01)	375 863	24 (<0.01)	619 338	−0.02 (−0.05 to 0.01)	0.40 (0.16 to 1.00)	.05
Other connective tissue diseases^f^	11 (<0.01)	375 859	8 (<0.01)	619 320	0.02 (−0.01 to 0.05)	2.44 (0.96 to 6.15)	.06
Vasculitis	22 (0.01)	375 772	26 (<0.01)	619 198	0.02 (−0.03 to 0.06)	1.35 (0.76 to 2.39)	.31
Inflammatory bowel disease	261 (0.07)	374 520	406 (0.07)	617 308	0.05 (−0.10 to 0.20)	1.08 (0.92 to 1.26)	.36
Spondyloarthropathies	44 (0.01)	375 711	74 (0.01)	619 113	−0.01 (−0.07 to 0.05)	0.93 (0.63 to 1.35)	.70
Psoriasis	121 (0.03)	374 902	203 (0.03)	618 085	−0.02 (−0.12 to 0.09)	0.95 (0.75 to 1.19)	.65
Bullous skin disorders	52 (0.01)	375 762	49 (0.01)	619 243	0.05 (−0.01 to 0.11)	1.57 (1.06 to 2.33)	.03
Autoimmune thyroid disease	106 (0.03)	375 426	170 (0.03)	618 744	−0.00 (−0.10 to 0.09)	0.98 (0.77 to 1.26)	.89

Abbreviations: AHR, adjusted hazard ratio; NA, not available.

^a^ Numbers in each subcohort for each specific autoimmune diagnosis do not add up to the original number of SARS-CoV-2 cases and controls because, for estimation of risks for each new-incident autoimmune diagnosis, a subcohort of individuals without history of the diagnosis in the past 5 years was constructed.

^b^ Excess burden greater than 0 denotes excess burden in a respective autoimmune diagnosis among infected cases vs controls.

^c^ AHR greater than 1 denotes higher risk of a respective autoimmune diagnosis among cases vs controls.

^d^ Each model was overlap weighted and regression adjusted based on demographic characteristics (age, sex, ethnicity), socioeconomic status (housing type), vaccination status (not fully vaccinated, fully vaccinated, fully vaccinated and received boosters), and comorbidities.

^e^ Risks could not be estimated due to too few numbers of a new-incident autoimmune diagnosis for that subcategory.

^f^ Included mixed connective tissue disease, Behçet disease, and polymyalgia rheumatica.

**Table 3.** Risks and Excess Burdens of Prespecified New-Incident Autoimmune Diagnoses in SARS-CoV-2 Cases, Stratified by Initial Infection Severity, vs Controls During Delta and Omicron BA.1 or BA.2 Variant Periods

Autoimmune diagnosis	Delta variant–predominance transmission^a^				Omicron BA.1 or BA.2 variant–predominance transmission^a^			
	Mild cases not requiring hospitalization vs controls (n = 93 865)		Hospitalized SARS-CoV-2 cases vs controls (n = 10 314)		Mild SARS-CoV-2 cases not requiring hospitalization vs controls (n = 368 385)		Hospitalized SARS-CoV-2 cases vs controls (n = 7518)	
	AHR (95% CI)^b^^,^^c^	Excess burden, weighted, per 1000 persons (95% CI)^d^	AHR (95% CI)^b^^,^^c^	Excess burden, weighted, per 1000 persons (95% CI)^d^	AHR (95% CI)^b^^,^^c^	Excess burden, weighted, per 1000 persons (95% CI)^d^	AHR (95% CI)^b^^,^^c^	Excess burden, weighted, per 1000 persons (95% CI)^d^
Systemic lupus erythematosus	0.19 (0.03 to 1.35)	−0.05 (−0.11 to 0.01)	1.94 (0.26 to 14.37)	0.06 (−0.25 to 0.37)	0.86 (0.54 to 1.35)	−0.01 (−0.07 to 0.04)	3.21 (0.94 to 10.95)	0.27 (−0.25 to 0.80)
Rheumatoid arthritis	0.79 (0.53 to 1.18)	−0.08 (−0.25 to 0.10)	0.81 (0.29 to 2.25)	−0.10 (−0.80 to 0.60)	1.16 (0.94 to 1.43)	0.06 (−0.06 to 0.17)	1.16 (0.47 to 2.88)	0.09 (−0.72 to 0.90)
Sjögren syndrome	0.69 (0.09 to 5.40)	−0.00 (−0.04 to 0.03)	NA^e^	NA^e^	1.84 (0.95 to 3.55)	0.02 (−0.01 to 0.06)	7.20 (1.56 to 33.21)	0.23 (−0.17 to 0.64)
Systemic sclerosis	NA^e^	NA^e^	NA^e^	NA^e^	1.14 (0.48 to 2.73)	0.00 (−0.02 to 0.03)	4.43 (0.44 to 44.18)	0.10 (−0.19 to 0.40)
Dermatomyositis or polymyositis	NA^e^	NA^e^	NA^e^	NA^e^	0.42 (0.17 to 1.03)	−0.02 (−0.05 to 0.01)	NA^e^	NA^e^
Other connective tissue diseases^f^	2.43 (0.51 to 11.62)	0.01 (−0.03 to 0.05)	NA^e^	NA^e^	2.44 (0.95 to 6.27)	0.02 (−0.01 to 0.04)	NA^e^	NA^e^
Vasculitis	0.39 (0.09 to 1.61)	−0.03 (−0.10 to 0.03)	NA^e^	NA^e^	1.21 (0.67 to 2.21)	0.01 (−0.03 to 0.05)	6.72 (1.44 to 31.31)	0.23 (−0.18 to 0.63)
Inflammatory bowel disease	0.84 (0.62 to 1.13)	−0.10 (−0.33 to 0.13)	1.34 (0.75 to 2.39)	0.40 (−0.77 to 1.57)	1.01 (0.86 to 1.19)	0.01 (−0.14 to 0.16)	2.23 (1.45 to 3.46)	1.78 (0.19 to 3.36)
Spondyloarthropathies	0.70 (0.32 to 1.54)	−0.03 (−0.12 to 0.06)	2.18 (0.63 to 7.55)	0.19 (−0.31 to 0.70)	0.82 (0.54 to 1.22)	−0.02 (−0.08 to 0.04)	2.54 (0.99 to 6.51)	0.45 (−0.29 to 1.19)
Psoriasis	0.83 (0.55 to 1.24)	−0.06 (−0.23 to 0.11)	0.71 (0.22 to 2.27)	−0.14 (−0.79 to 0.51)	0.94 (0.74 to 1.18)	−0.02 (−0.13 to 0.08)	1.75 (0.76 to 4.04)	0.34 (−0.48 to 1.15)
Bullous skin disorders	0.20 (0.05 to 0.84)	−0.08 (−0.16–0.00)	2.14 (0.97 to 4.72)	0.48 (−0.33 to 1.30)	1.15 (0.74 to 1.79)	0.01 (−0.04 to 0.07)	4.88 (2.47 to 9.66)	1.51 (0.41 to 2.62)
Autoimmune thyroid disease	0.95 (0.58 to 1.55)	−0.01 (−0.15 to 0.13)	0.66 (0.16 to 2.75)	−0.12 (−0.67 to 0.43)	0.97 (0.75 to 1.24)	−0.01 (−0.11 to 0.09)	1.52 (0.59 to 3.89)	0.23 (−0.54 to 1.01)

Abbreviations: AHR, adjusted hazard ratio; NA, not available.

^a^ SARS-CoV-2 cases were stratified by severity of initial infection (mild cases managed in ambulatory care alone vs cases requiring hospitalization). Results for the subgroup of individuals with severe infection (requiring oxygen or intensive care unit/high-dependency admission) were not presented because risks of individual autoimmune diagnoses could not be estimated due to too few cases; however, there was no increased risk of any autoimmune diagnosis (composite outcome) in severe cases compared with controls during Delta (AHR, 1.40; 95% CI, 0.86-2.31) and Omicron (AHR, 1.65; 95% CI, 1.00-2.71) variant periods.

^b^ AHR greater than 1 denotes higher risk of a respective autoimmune diagnosis among cases vs controls.

^c^ Each model was overlap weighted and regression adjusted based on demographic characteristics (age, sex, ethnicity), socioeconomic status (housing type), and comorbidities.

^d^ Excess burden greater than 0 denotes excess burden in a respective autoimmune diagnosis among infected cases vs controls.

^e^ Risks could not be estimated due to too few numbers of a new-incident autoimmune diagnosis for that subcategory.

^f^ Included mixed connective tissue disease, Behçet disease, and polymyalgia rheumatica.

**Table 4.** Risks and Excess Burdens of Prespecified New-Incident Autoimmune Diagnoses in SARS-CoV-2 Cases vs Controls, Stratified by Vaccination Status, During Delta and Omicron BA.1 or BA.2 Variant Periods

Autoimmune diagnosis	Delta variant–predominance transmission				Omicron BA.1 or BA.2 variant–predominance transmission			
	Fully vaccinated cases vs controls (n = 84 518)^a^		Received boosters cases vs controls (n = 11 059)^a^		Fully vaccinated cases vs controls (n = 83 506)^a^		Received boosters cases vs controls (n = 280 254)^a^	
	AHR (95% CI)^b^^,^^c^	Excess burden weighted, per 1000 persons (95% CI)^d^	AHR (95% CI)^b^^,^^c^	Excess burden, weighted, per 1000 persons (95% CI)^d^	AHR (95% CI)^b^^,^^c^	Excess burden, weighted, per 1000 persons (95% CI)^d^	AHR (95% CI)^b^^,^^c^	Excess burden, weighted, per 1000 persons (95% CI)^d^
Systemic lupus erythematosus	NA^e^	NA^e^	NA^e^	NA^e^	1.27 (0.63 to 2.56)	0.03 (−0.10 to 0.16)	0.78 (0.44 to 1.38)	−0.02 (−0.08 to 0.04)
Rheumatoid arthritis	0.81 (0.53 to 1.24)	−0.07 (−0.26 to 0.12)	0.34 (0.08 to 1.39)	−0.38 (−0.93 to 0.18)	1.44 (0.94 to 2.22)	0.12 (−0.09 to 0.33)	1.07 (0.84 to 1.37)	0.03 (−0.11 to 0.17)
Sjögren syndrome	0.63 (0.08 to 5.07)	−0.01 (−0.05 to 0.03)	NA^e^	NA^e^	NA^e^	NA^e^	1.91 (0.92 to 3.96)	0.03 (−0.02 to 0.08)
Systemic sclerosis	NA^e^	NA^e^	NA^e^	NA^e^	NA^e^	NA^e^	0.91 (0.35 to 2.38)	−0.00 (−0.04 to 0.03)
Dermatomyositis or polymyositis	NA^e^	NA^e^	NA^e^	NA^e^	NA^e^	NA^e^	0.49 (0.19 to 1.26)	−0.02 (−0.06 to 0.02)
Other connective tissue diseases^f^	NA^e^	NA^e^	NA^e^	NA^e^	NA^e^	NA^e^	3.38 (0.87 to 13.11)	0.02 (−0.01 to 0.04)
Vasculitis	0.21 (0.03 to 1.57)	−0.05 (−0.11 to 0.02)	NA^e^	NA^e^	5.74 (1.48 to 22.23)	0.07 (0.01 to 0.14)	0.96 (0.49 to 1.88)	−0.00 (−0.05 to 0.05)
Inflammatory bowel disease	0.97 (0.72 to 1.31)	−0.02 (−0.28 to 0.24)	0.89 (0.43 to 1.86)	−0.09 (−0.87 to 0.70)	1.15 (0.85 to 1.55)	0.10 (−0.21 to 0.41)	1.02 (0.85 to 1.24)	0.02 (−0.16 to 0.19)
Spondyloarthropathies	0.99 (0.48 to 2.04)	−0.00 (−0.10 to 0.10)	0.61 (0.08 to 4.76)	−0.06 (−0.37 to 0.25)	0.59 (0.25 to 1.36)	−0.06 (−0.18 to 0.06)	1.01 (0.64 to 1.60)	0.00 (−0.07 to 0.08)
Psoriasis	0.89 (0.59 to 1.34)	−0.04 (−0.23 to 0.15)	0.51 (0.12 to 2.14)	−0.18 (−0.65 to 0.29)	1.01 (0.64 to 1.61)	0.00 (−0.20 to 0.20)	0.88 (0.68 to 1.16)	−0.04 (−0.17 to 0.09)
Bullous skin disorders	0.80 (0.35 to 1.80)	−0.02 (−0.12 to 0.08)	0.48 (0.06 to 3.76)	−0.09 (−0.41 to 0.23)	2.08 (0.99 to 4.35)	0.08 (−0.04 to 0.21)	1.26 (0.77 to 2.08)	0.02 (−0.05 to 0.09)
Autoimmune thyroid disease	0.97 (0.57 to 1.65)	−0.01 (−0.15 to 0.14)	0.88 (0.20 to 3.79)	−0.03 (−0.43 to 0.38)	1.03 (0.61 to 1.75)	0.01 (−0.17 to 0.18)	0.99 (0.75 to 1.32)	−0.00 (−0.12 to 0.12)

Abbreviations: AHR, adjusted hazard ratio; mRNA, messenger RNA; NA, not available.

^a^ Fully vaccinated status (ie, completed primary vaccination series only) was defined as receiving 2 doses of an mRNA COVID-19 vaccine at least 8 weeks apart. Booster status (ie, received 1 or more booster vaccination doses) was defined as receiving at least a third dose of an mRNA COVID-19 vaccine 6 to 9 months after the second dose. Results for the subgroup of individuals who did not complete primary vaccination series (ie, either unvaccinated or partially vaccinated with a single vaccine dose of an mRNA COVID-19 vaccine) were not presented because risks of individual autoimmune diagnoses could not be estimated due to too few cases; however, there was no increased risk of any autoimmune diagnosis (composite outcome) in unvaccinated or partially vaccinated cases, compared with controls, during the Delta (AHR, 0.70; 95% CI, 0.46-1.06) and Omicron (AHR, 1.28; 95% CI, 0.82-1.99) variant periods.

^b^ AHR greater than 1 denotes higher risk of a respective autoimmune diagnosis among cases vs controls.

^c^ Each model was overlap weighted and regression adjusted based on demographic characteristics (age, sex, ethnicity), socioeconomic status (housing type), and comorbidities.

^d^ Excess burden greater than 0 denotes excess burden in a respective autoimmune diagnosis among infected cases vs controls.

^e^ Risks could not be estimated due to too few numbers of new-incident autoimmune diagnosis for that subcategory.

^f^ Included mixed connective tissue disease, Behcet disease, and polymyalgia rheumatica.

Estimates of risk were robust to sensitivity analyses using inverse propensity weighting instead of overlap weighting (eTables 5-7 in Supplement 1). No significantly elevated risk of new-incident autoimmune sequelae was observed across subgroup analyses by sex, age, and ethnicity (eTables 8-14 in Supplement 1). There was also no significantly increased risk of controls with negative outcomes (eTable 15 in Supplement 1).

## Discussion

In a cohort of cases largely vaccinated against COVID-19 who also received boosters, there was no significantly increased risk of any autoimmune sequelae up to 300 days after infection during the predominance of the Delta and Omicron BA.1 or BA.2 variants, compared with controls. An exception was an increased risk of Sjögren syndrome and bullous skin disorders after Omicron variant infection, which did not cross the threshold of significance when adjusted for multiple comparisons.

These findings substantially differ from the majority of studies that have reported increased long-term incidence of a wide range of autoimmune sequelae after SARS-CoV-2 infection, albeit from waves of earlier SARS-CoV-2 variants^3,4,5,9,10^ and without accounting for the potential protection from COVID-19 vaccination,^3,4,5,8^ including boosters. For instance, in a large study that examined the risks of postacute autoimmune sequelae in the pre-Omicron variant era across an unvaccinated population drawn from 48 US-based health care organizations, elevated risks of a wide range of autoimmune conditions, including Sjögren syndrome, rheumatoid arthritis, spondyloarthropathies, and psoriasis (AHR range, 2.32-3.21) were observed after COVID-19 compared with controls.^3^ In a large EHR-based cohort study including approximately 4 million patients, fewer than 10% of whom were vaccinated, risk of any autoimmune disease was lower during the predominance of the Omicron variant.^6^ Similarly, in a Hong Kong population with SARS-CoV-2 infection during the Omicron variant period (81.9% of whom were vaccinated with 2 doses), the AHRs of new-incident autoimmune conditions after infection were generally lower than estimates from the pre-Omicron variant studies.^7^

In the present cohort, three-quarters of whom had received an additional third vaccine dose (booster) prior to infection with the Omicron variant, significantly elevated risks of inflammatory bowel disease and bullous skin disorders were observed only in the subset of cases requiring hospitalization during the predominance of the Omicron variant; no significantly elevated risk of autoimmune disease was observed after infection with the Delta variant. These differences may be associated with the protective properties of vaccination and booster doses against severe COVID-19 in this highly vaccinated cohort. Increased risk of autoimmune sequelae after infection with pre-Omicron variant has been attributed to differences in the severity of the initial infection.^6^ However, the majority of the population in Singapore was already vaccinated or had already received a booster prior to infection and had only mild infection that did not require hospitalization.^20^ In nonhospitalized adults, SARS-CoV-2 Delta or Omicron variant infection had similar illness duration, symptom severity, and viral kinetics regardless of variant type.^21^ In a hospitalized cohort, an Omicron variant infection showed faster viral replication compared with a Delta variant infection^22^; escalating replicative fitness has been reported with successive Omicron variants.^23^ Replicative fitness, in turn, may be associated with viral persistence. SARS-CoV-2 persistence in tissues has been associated with increased likelihood of developing post–COVID-19 condition symptoms^24^ and is hypothesized to play a role in autoimmunity via generation of cross-reactive antibodies targeted against widely distributed SARS-CoV-2 antigens within tissues.^10^ Continued surveillance for autoimmune conditions arising after COVID-19 is still necessary during the Omicron variant era, although the burden of new-incident postacute autoimmune sequelae may be less substantial than originally anticipated.

Significantly elevated risks of inflammatory bowel disease and bullous skin disorders were observed only in the subset of COVID-19 cases requiring hospitalization for the Omicron variant infection, compared with controls. Similarly, in population-based cohorts from Korea and Germany who contracted SARS-CoV-2 infection before the predominance of the Omicron variant, overall risks of new-incident autoimmune sequelae increased markedly with the severity of the acute stage of COVID-19.^5,10^ Increased risk of inflammatory bowel disease after COVID-19 was reported in population-based cohorts from the UK, Japan, Korea, and the US who contracted SARS-CoV-2 infection before the predominance of the Omicron variant,^3,4,5,10^ while increased risk of pemphigoid (AHR, 2.39; 95% CI, 1.83-3.11) was reported after Omicron variant infection in Hong Kong adults.^7^ Prolonged inflammation provoked by COVID-19 may trigger the immune system to generate antibodies against virus antigens that cross-react with structurally similar self-antigens widely distributed in skin and other connective tissues.^25^ Chronic persistence of viral antigen in gut mucosal tissue approximately 7 months after COVID-19 was documented in a cohort of individuals who developed inflammatory bowel disease, with postacute sequelae reported from the majority of patients with viral antigen persistence but not in patients without.^26^ Hyperstimulation and persistence of a proinflammatory state induced by SARS-CoV-2 infection may also be associated with dysregulation of inflammation and immune response,^27^ contributing to loss of tolerance to self-antigens. Autoantibodies were identified in approximately 50% of patients with COVID-19 but in fewer than 15% of healthy controls.^1^ Increased autoantibody production was associated with greater severity of acute illness.^12,28,29,30^ Severe illness requiring oxygen supplementation was associated with subsequent development of autoantibodies.^28^ In a prospective case-control study, 15% to 45% of cases admitted to the intensive care unit had positive result for autoantibodies compared with only 2 cases in the outpatient group.^29^ Similarly, 2-fold higher titers of anti–angiotensin-converting enzyme antibodies were reported in patients with severe infection compared with asymptomatic cases.^11^ Results positive for persistence of autoantibodies at 12 months after COVID-19 were, in turn, associated with nonresolving symptoms (eg, fatigue) and elevated levels of proinflammatory cytokines in a subset of COVID-19 survivors,^2^ with positive antinuclear antibody results associated with increased risk of new-incident autoimmune disease after COVID-19.^6^ Additional studies are required to better elucidate the pathophysiological mechanism underlying new-onset autoimmune sequelae after SARS-CoV-2 infection. However, the burden of new-onset autoimmune sequelae after Omicron variant infection is anticipated to be low and confined to the small subset of cases with more severe disease.

Clinical evidence is mixed regarding the protective properties of vaccination against risk of autoimmune sequelae after SARS-CoV-2 infection. In a large EHR study across 74 health care organizations that extended into the period of Omicron variant predominance and included almost 4 million patients, vaccination (vs no vaccination) was associated with an 18% increased risk of a new-incident autoimmune disease after infection; however, only 8.2% of patients were vaccinated, which was likely a substantial underestimation.^6^ In contrast, in a cohort of over 10 million Korean adults with infection during the predominance of the pre-Delta and Delta variants, receipt of 1 or more vaccine doses was associated with reduced risk for new-incident autoimmune disease after mild but not moderate-to-severe COVID-19, although further breakdown of vaccination status by booster and nonbooster groups was unavailable.^9^ Similarly, completion of a primary vaccination series attenuated the risk of new-incident autoimmune sequelae after SARS-CoV-2-infection in the Omicron and pre-Omicron variant era in population-based cohorts in Hong Kong and Korea, respectively^7,10^; however, 45% of the Korean cohort remained unvaccinated,^10^ and 55% of the Hong Kong cohort had yet to receive a third vaccine dose. In the highly vaccinated population of Singapore, while elevated risk of vasculitis was observed with vaccine-breakthrough Omicron variant infections compared with controls, this outcome was attenuated by booster vaccination; no increased risk of vasculitis was observed in the subgroup who received boosters. The results support the continued enrollment of at-risk individuals for booster vaccinations during COVID-19 endemicity. Given persistent vaccine hesitancy, highlighting benefits may help increase acceptance,^30^ particularly as COVID-19 vaccination is likely to be protective against autoimmunity, rather than contributory. In the Singaporean population, severe flares of autoimmune disease after COVID-19 vaccination were rare in individuals with preexisting autoimmune conditions.^31^

### Strengths and Limitations

The strengths of this study include use of comprehensive nationwide registries to classify SARS-CoV-2 infection and vaccination status to investigate the incidence of autoimmune diseases after COVID-19 in a highly vaccinated population. New-incident autoimmune diseases were identified from a comprehensive health care claims database with nationwide coverage, minimizing selection bias caused by loss to follow-up. A large number of potential confounders were controlled for, and the analyses took into account adjustment for multiple testing, thereby reducing the likelihood of false-positive findings. A test-negative design was also used, reducing bias associated with confounding by health care–seeking behavior.

This study has several limitations. First, while comprehensive nationwide registries were used to classify SARS-CoV-2 infection status, control groups may still be contaminated by undiagnosed or asymptomatic infections, although mandatory testing at health care settings and provision of free testing were put in place to mitigate this issue. Misclassification of exposure would bias estimated HRs and excess burdens downward. Second, variant was imputed according to the period of predominant transmission and not individual-level sequencing. Third, administrative claims data were used to capture autoimmune diagnoses, while comprehensive, additional corroborative information, such as laboratory tests for autoantibodies, was unavailable. Fourth, for some individual autoimmune sequelae, rarity resulted in only a small number of new-incident cases; therefore, estimates could not be provided across all subgroups and could have resulted in imprecise interpretations. Further research is needed to replicate these findings in other populations and to measure autoantibody profiles in prospective cohorts of individuals with SARS-CoV-2 infection.

## Conclusions

In this retrospective cohort study of vaccinated adults in Singapore who also received boosters against COVID-19, no significantly elevated long-term risk of autoimmune sequelae was observed after Delta and Omicron BA.1 or BA.2 variant infection except for a modestly increased risk of inflammatory bowel disease and bullous skin disorders in the hospitalized subgroup during the predominance of the Omicron variant. Booster vaccination appeared to mitigate the risk of long-term autoimmune sequelae.

## Notes

Supplement 1. **eTable 1.** Risks and Excess Burdens of Pre-specified New-Incident Autoimmune Diagnoses in Mild Infected Cases Not Requiring Hospitalisation and Test-Negative Control Groups During Delta and Omicron BA.1/2 Periods **eTable 2.** Risks and Excess Burdens of Pre-specified New-Incident Autoimmune Diagnoses in Infected Hospitalised Cases and Test-Negative Control Groups During Delta and Omicron BA.1/2 Periods **eTable 3.** Risks and Excess Burdens of Pre-specified New-Incident Autoimmune Diagnoses in Infected Cases and Test-Negative Control Groups During Delta and Omicron BA.1/2 Periods, Completed Full Primary Vaccination Series **eTable 4.** Risks and Excess Burdens of Pre-specified New-Incident Autoimmune Diagnoses in Infected Cases and Test-Negative Control Groups During Delta and Omicron BA.1/2 Periods, Boosted **eTable 5.** Risks and Excess Burdens of Pre-specified New-Incident Autoimmune Diagnoses in Infected Cases and Test-Negative Control Groups During Delta and Omicron BA.1/2 Periods, Using Inverse Propensity Weighting **eTable 6.** Risks and Excess Burdens of Pre-specified New-Incident Autoimmune Diagnoses in Infected Cases Test-Negative Control Groups During Delta and Omicron BA.1/2 Periods Stratified by Initial Infection Severity, Using Inverse Propensity Weighting **eTable 7.** Risks and Excess Burdens of Pre-specified New-Incident Autoimmune Diagnoses in Infected Cases and Test-Negative Control Groups During Delta and Omicron BA.1/2 Periods Stratified by Vaccination Status, Using Inverse Propensity Weighting **eTable 8.** Risks and Excess Burdens of Pre-specified New-Incident Autoimmune Diagnoses in Infected Cases and Test-Negative Control Groups During Delta and Omicron BA.1/2 Periods (Male Subgroup) **eTable 9.** Risks and Excess Burdens of Pre-specified New-Incident Autoimmune Diagnoses in Infected Cases and Test-Negative Control Groups During Delta and Omicron BA.1/2 Periods (Female Subgroup) **eTable 10.** Risks and Excess Burdens of Pre-specified New-Incident Autoimmune Diagnoses in Infected Cases and Test-Negative Control Groups During Delta and Omicron BA.1/2 Periods (Age 18-64 Years Subgroup) **eTable 11.** Risks and Excess Burdens of Pre-specified New-Incident Autoimmune Diagnoses in Infected Cases and Test-Negative Control Groups During Delta and Omicron BA.1/2 Periods (Age ≥65 Years Subgroup) **eTable 12.** Risks and Excess Burdens of Pre-specified New-Incident Autoimmune Diagnoses in Infected Cases and Test-Negative Control Groups During Delta and Omicron BA.1/2 Periods (Chinese Subgroup) **eTable 13.** Risks and Excess Burdens of Pre-specified New-Incident Autoimmune Diagnoses in Infected Cases and Test-Negative Control Groups During Delta and Omicron BA.1/2 Periods (Malay Subgroup) **eTable 14.** Risks and Excess Burdens of Pre-specified New-Incident Autoimmune Diagnoses in Infected Cases and Test-Negative Control Groups During Delta and Omicron BA.1/2 Periods (Indian Subgroup) **eTable 15.** Risks and Excess Burdens of Pre-specified Negative Outcome Controls in Infected Cases vs Test-Negative Control Groups During Delta and Omicron BA.1/2 Periods **eAppendix.** List of ICD-10 Codes Used for Outcomes of Interest

Supplement 2.
Data Sharing Statement
