# Supplementary material for: Autoimmune Sequelae After Delta or Omicron Variant SARS-CoV-2 Infection in a Highly Vaccinated Cohort
Source: JAMA Netw Open. 2024 Aug 30;7(8):e2430983. doi: 10.1001/jamanetworkopen.2024.30983 (PMC11364997; doi:10.1001/jamanetworkopen.2024.30983)
Supplement: Supplement 1. — eTable 1. Risks and Excess Burdens of Pre-specified New-Incident Autoimmune Diagnoses in Mild Infected Cases Not Requiring Hospitalisation and Test-Negative Control Groups During Delta and Omicron BA.1/2 Periods eTable 2. Risks and Excess Burdens of Pre-specified New-Incident Autoimmune Diagnoses in Infected Hospitalised Cases and Test-Negative Control Groups During Delta and Omicron BA.1/2 Periods eTable 3. Risks and Excess Burdens of Pre-specified New-Incident Autoimmune Diagnoses in Infected Cases and Test-Negative Control Groups During Delta and Omicron BA.1/2 Periods, Completed Full Primary Vaccination Series eTable 4. Risks and Excess Burdens of Pre-specified New-Incident Autoimmune Diagnoses in Infected Cases and Test-Negative Control Groups During Delta and Omicron BA.1/2 Periods, Boosted eTable 5. Risks and Excess Burdens of Pre-specified New-Incident Autoimmune Diagnoses in Infected Cases and Test-Negative Control Groups During Delta and Omicron BA.1/2 Periods, Using Inverse Propensity Weighting eTable 6. Risks and Excess Burdens of Pre-specified New-Incident Autoimmune Diagnoses in Infected Cases Test-Negative Control Groups During Delta and Omicron BA.1/2 Periods Stratified by Initial Infection Severity, Using Inverse Propensity Weighting eTable 7. Risks and Excess Burdens of Pre-specified New-Incident Autoimmune Diagnoses in Infected Cases and Test-Negative Control Groups During Delta and Omicron BA.1/2 Periods Stratified by Vaccination Status, Using Inverse Propensity Weighting eTable 8. Risks and Excess Burdens of Pre-specified New-Incident Autoimmune Diagnoses in Infected Cases and Test-Negative Control Groups During Delta and Omicron BA.1/2 Periods (Male Subgroup) eTable 9. Risks and Excess Burdens of Pre-specified New-Incident Autoimmune Diagnoses in Infected Cases and Test-Negative Control Groups During Delta and Omicron BA.1/2 Periods (Female Subgroup) eTable 10. Risks and Excess Burdens of Pre-specified New-Incident Autoimmune Diagnoses in Infec [file jamanetwopen-e2430983-s001.pdf]

Supplemental Online Content

Wee LE, Lim JT, Tay AT, et al. Autoimmune sequelae post–Delta or Omicron variant SARS-CoV-2 infection in a highly vaccinated cohort. *JAMA Netw Open*. 2024;7(8):e2430983. doi:10.1001/jamanetworkopen.2024.30983

eTable 1. Risks and Excess Burdens of Pre-specified New-Incident Autoimmune Diagnoses in Mild Infected Cases Not Requiring Hospitalisation and Test-Negative Control Groups During Delta and Omicron BA.1/2 Periods

eTable 2. Risks and Excess Burdens of Pre-specified New-Incident Autoimmune Diagnoses in Infected Hospitalised Cases and Test-Negative Control Groups During Delta and Omicron BA.1/2 Periods

eTable 3. Risks and Excess Burdens of Pre-specified New-Incident Autoimmune Diagnoses in Infected Cases and Test-Negative Control Groups During Delta and Omicron BA.1/2 Periods, Completed Full Primary Vaccination Series

eTable 4. Risks and Excess Burdens of Pre-specified New-Incident Autoimmune Diagnoses in Infected Cases and Test-Negative Control Groups During Delta and Omicron BA.1/2 Periods, Boosted

eTable 5. Risks and Excess Burdens of Pre-specified New-Incident Autoimmune Diagnoses in Infected Cases and Test-Negative Control Groups During Delta and Omicron BA.1/2 Periods, Using Inverse Propensity Weighting

eTable 6. Risks and Excess Burdens of Pre-specified New-Incident Autoimmune Diagnoses in Infected Cases Test-Negative Control Groups During Delta and Omicron BA.1/2 Periods Stratified by Initial Infection Severity, Using Inverse Propensity Weighting

eTable 7. Risks and Excess Burdens of Pre-specified New-Incident Autoimmune Diagnoses in Infected Cases and Test-Negative Control Groups During Delta and Omicron BA.1/2 Periods Stratified by Vaccination Status, Using Inverse Propensity Weighting

eTable 8. Risks and Excess Burdens of Pre-specified New-Incident Autoimmune Diagnoses in Infected Cases and Test-Negative Control Groups During Delta and Omicron BA.1/2 Periods (Male Subgroup)

eTable 9. Risks and Excess Burdens of Pre-specified New-Incident Autoimmune Diagnoses in Infected Cases and Test-Negative Control Groups During Delta and Omicron BA.1/2 Periods (Female Subgroup)

© 2024 Wee LE et al. *JAMA Network Open*

eTable 10. Risks and Excess Burdens of Pre-specified New-Incident Autoimmune Diagnoses in Infected Cases and Test-Negative Control Groups During Delta and Omicron BA.1/2 Periods (Age 18-64 Years Subgroup)

eTable 11. Risks and Excess Burdens of Pre-specified New-Incident Autoimmune Diagnoses in Infected Cases and Test-Negative Control Groups During Delta and Omicron BA.1/2 Periods (Age ≥65 Years Subgroup)

eTable 12. Risks and Excess Burdens of Pre-specified New-Incident Autoimmune Diagnoses in Infected Cases and Test-Negative Control Groups During Delta and Omicron BA.1/2 Periods (Chinese Subgroup)

eTable 13. Risks and Excess Burdens of Pre-specified New-Incident Autoimmune Diagnoses in Infected Cases and Test-Negative Control Groups During Delta and Omicron BA.1/2 Periods (Malay Subgroup)

eTable 14. Risks and Excess Burdens of Pre-specified New-Incident Autoimmune Diagnoses in Infected Cases and Test-Negative Control Groups During Delta and Omicron BA.1/2 Periods (Indian Subgroup)

eTable 15. Risks and Excess Burdens of Pre-specified Negative Outcome Controls in Infected Cases vs Test-Negative Control Groups During Delta and Omicron BA.1/2 Periods

eAppendix. List of ICD-10 Codes Used for Outcomes of Interest

This supplemental material has been provided by the authors to give readers additional information about their work.

eTable 1: Risks and excess burdens of pre-specified new-incident autoimmune diagnoses in mild infected cases not requiring hospitalisation and test-negative control groups during Delta and Omicron BA.1/2 periods

New-incident autoimmune diagnoses in mild infected cases not requiring hospitalisation and test-negative control groups during Delta period

**Autoimmune diagnoses:**	Cases with diagnosis N(%)[a]	Infected cases (N)[a]	Controls with diagnosis N(%)[a]	Test-negative controls (N)[a]	Excess burden (weighted, per 1000 persons), 95% CI	Adjusted hazards ratio(aHR),[c] 95% CI
Systemic lupus erythematosus	1(0.00)	93880	47(0.01)	666367	-0.05 (-0.11-0.01)	0.19(0.03-1.35)
Rheumatoid arthritis	27(0.03)	93706	250(0.04)	665098	-0.08 (-0.25-0.10)	0.79(0.53-1.18)
Sjogren's syndrome	1(0.00)	93897	11(0.00)	666463	-0.00 (-0.04-0.03)	0.69(0.09-5.40)
Systemic sclerosis	1(0.00)	93905	6(0.00)	666535	-#	-#
Dermatomyositis/polymyositis	0(0.00)	93904	28(0.00)	666515	-#	-#
Other connective tissue diseases [b]	2(0.00)	93903	8(0.00)	666531	0.01 (-0.03-0.05)	2.43(0.51-11.62)
Vasculitis	2(0.00)	93885	37(0.01)	666363	-0.03 (-0.10-0.03)	0.39(0.09-1.61)
Inflammatory bowel disease	48(0.05)	93637	422(0.06)	664169	-0.10 (-0.33-0.13)	0.84(0.62-1.13)
Spondyloarthropathies	7(0.01)	93868	64(0.01)	666265	-0.03 (-0.12-0.06)	0.70(0.32-1.54)
Psoriasis	27(0.03)	93658	210(0.03)	665114	-0.06 (-0.23-0.11)	0.83(0.55-1.24)
Bullous skin disorders	2(0.00)	93883	62(0.01)	666401	-0.08 (-0.16--0.00)	0.20(0.05-0.84)
Autoimmune thyroid disease	18(0.02)	93808	142(0.02)	665825	-0.01 (-0.15-0.13)	0.95(0.58-1.55)

New-incident autoimmune diagnoses in mild infected cases not requiring hospitalisation and test-negative control groups during Omicron BA.1/2 period

**Autoimmune diagnoses:**	Cases with diagnosis N(%)[a]	Infected cases (N)[a]	Controls with diagnosis N(%)[a]	Test-negative controls (N)[a]	Excess burden (weighted, per 1000 persons), 95% CI	Adjusted hazards ratio(aHR),[c] 95% CI
Systemic lupus erythematosus	28(0.01)	366446	58(0.01)	619189	-0.01 (-0.07-0.04)	0.86(0.54-1.35)
Rheumatoid arthritis	146(0.04)	365685	221(0.04)	618087	0.06 (-0.06-0.17)	1.16(0.94-1.43)
Sjogren's syndrome	19(0.01)	366533	18(0.00)	619290	0.02 (-0.01-0.06)	1.84(0.95-3.55)
Systemic sclerosis	9(0.00)	366567	12(0.00)	619348	0.00 (-0.02-0.03)	1.14(0.48-2.73)
Dermatomyositis/polymyositis	6(0.00)	366559	24(0.00)	619338	-0.02 (-0.05-0.01)	0.42(0.17-1.03)
Other connective tissue diseases [b]	10(0.00)	366555	8(0.00)	619320	0.02 (-0.01-0.04)	2.44(0.95-6.27)
Vasculitis	19(0.01)	366475	26(0.00)	619198	0.01 (-0.03-0.05)	1.21(0.67-2.21)
Inflammatory bowel disease	232(0.06)	365344	406(0.07)	617308	0.01 (-0.14-0.16)	1.01(0.86-1.19)
Spondyloarthropathies	36(0.01)	366421	74(0.01)	619113	-0.02 (-0.08-0.04)	0.82(0.54-1.22)
Psoriasis	115(0.03)	365642	203(0.03)	618085	-0.02 (-0.13-0.08)	0.94(0.74-1.18)
Bullous skin disorders	34(0.01)	366498	49(0.01)	619243	0.01 (-0.04-0.07)	1.15(0.74-1.79)
Autoimmune thyroid disease	100(0.03)	366146	170(0.03)	618744	-0.01 (-0.11-0.09)	0.97(0.75-1.24)

HR > 1 denotes higher risk of a respective autoimmune diagnosis amongst infected cases, versus test-negative control group

Excess burden > 0 denotes excess burden in a respective autoimmune diagnosis amongst infected cases, versus test-negative control group

[a] Numbers in each subcohort for each specific autoimmune diagnosis do not add up to the original number of infected cases and test-negative controls because for estimation of risks for each new-incident autoimmune diagnosis, a sub-cohort of individuals without history of the diagnosis in the past 5 years was constructed.

[b] Other connective tissue diseases included: mixed connective tissue disease; Behcet's disease; polymyalgia rheumatica

[c] Each model is overlap weighted and regression adjusted based on demographic characteristics (age, sex, ethnicity), socioeconomic status (housing type), vaccination status (not fully vaccinated, fully vaccinated, fully vaccinated and boosted), and comorbidities

[#] Risks could not be estimated due to too few numbers of new-incident autoimmune diagnosis for that subcategory.

© 2024 Wee LE et al. *JAMA Network Open*

eTable 2: Risks and excess burdens of pre-specified new-incident autoimmune diagnoses in infected hospitalised cases and test-negative control groups during Delta and Omicron BA.1/2 periods

New-incident autoimmune diagnoses in infected hospitalised cases and test-negative control groups during Delta period

**Autoimmune diagnoses:**	Cases with diagnosis N(%)[a]	Infected cases (N)[a]	Controls with diagnosis N(%)[a]	Test-negative controls (N)[a]	Excess burden (weighted, per 1000 persons), 95% CI	Adjusted hazards ratio(aHR),[c] 95% CI
Systemic lupus erythematosus	1(0.01)	8261	47(0.01)	666367	0.06 (-0.25-0.37)	1.94(0.26-14.37)
Rheumatoid arthritis	4(0.05)	8230	250(0.04)	665098	-0.10 (-0.80-0.60)	0.81(0.29-2.25)
Sjogren's syndrome	0(0.00)	8276	11(0.00)	666463	-#	-#
Systemic sclerosis	0(0.00)	8275	6(0.00)	666535	-#	-#
Dermatomyositis/polymyositis	0(0.00)	8276	28(0.00)	666515	-#	-#
Other connective tissue diseases [b]	0(0.00)	8274	8(0.00)	666531	-#	-#
Vasculitis	0(0.00)	8269	37(0.01)	666363	-#	-#
Inflammatory bowel disease	13(0.16)	8190	422(0.06)	664169	0.40 (-0.77-1.57)	1.34(0.75-2.39)
Spondyloarthropathies	3(0.04)	8270	64(0.01)	666265	0.19 (-0.31-0.70)	2.18(0.63-7.55)
Psoriasis	3(0.04)	8241	210(0.03)	665114	-0.14 (-0.79-0.51)	0.71(0.22-2.27)
Bullous skin disorders	8(0.10)	8265	62(0.01)	666401	0.48 (-0.33-1.30)	2.14(0.97-4.72)
Autoimmune thyroid disease	2(0.02)	8253	142(0.02)	665825	-0.12 (-0.67-0.43)	0.66(0.16-2.75)

New-incident autoimmune diagnoses in infected hospitalised cases and test-negative control groups during Omicron BA.1/2 period

**Autoimmune diagnoses:**	Cases with diagnosis N(%)[a]	Infected cases (N)[a]	Controls with diagnosis N(%)[a]	Test-negative controls (N)[a]	Excess burden (weighted, per 1000 persons), 95% CI	Adjusted hazards ratio(aHR),[c] 95% CI
Systemic lupus erythematosus	3(0.04)	7889	58(0.01)	619189	0.27 (-0.25-0.80)	3.21(0.94-10.95)
Rheumatoid arthritis	5(0.06)	7854	221(0.04)	618087	0.09 (-0.72-0.90)	1.16(0.47-2.88)
Sjogren's syndrome	2(0.03)	7912	18(0.00)	619290	0.23 (-0.17-0.64)	7.20(1.56-33.21)
Systemic sclerosis	1(0.01)	7918	12(0.00)	619348	0.10 (-0.19-0.40)	4.43(0.44-44.18)
Dermatomyositis/polymyositis	0(0.00)	7915	24(0.00)	619338	-#	-#
Other connective tissue diseases [b]	1(0.01)	7916	8(0.00)	619320	-#	-#
Vasculitis	2(0.03)	7912	26(0.00)	619198	0.23 (-0.18-0.63)	6.72(1.44-31.31)
Inflammatory bowel disease	27(0.35)	7809	406(0.07)	617308	1.78 (0.19-3.36)	2.23(1.45-3.46)
Spondyloarthropathies	6(0.08)	7907	74(0.01)	619113	0.45 (-0.29-1.19)	2.54(0.99-6.51)
Psoriasis	6(0.08)	7879	203(0.03)	618085	0.34 (-0.48-1.15)	1.75(0.76-4.04)
Bullous skin disorders	15(0.19)	7888	49(0.01)	619243	1.51 (0.41-2.62)	4.88(2.47-9.66)
Autoimmune thyroid disease	5(0.06)	7895	170(0.03)	618744	0.23 (-0.54-1.01)	1.52(0.59-3.89)

HR > 1 denotes higher risk of a respective autoimmune diagnosis amongst infected cases, versus test-negative control group

Excess burden > 0 denotes excess burden in a respective autoimmune diagnosis amongst infected cases, versus test-negative control group

[a] Numbers in each subcohort for each specific autoimmune diagnosis do not add up to the original number of infected cases and test-negative controls because for estimation of risks for each new-incident autoimmune diagnosis, a sub-cohort of individuals without history of the diagnosis in the past 5 years was constructed.

[b] Other connective tissue diseases included: mixed connective tissue disease; Behcet's disease; polymyalgia rheumatica

[c] Each model is overlap weighted and regression adjusted based on demographic characteristics (age, sex, ethnicity), socioeconomic status (housing type), vaccination status (not fully vaccinated, fully vaccinated, fully vaccinated and boosted), and comorbidities

[#] Risks could not be estimated due to too few numbers of new-incident autoimmune diagnosis for that subcategory.

© 2024 Wee LE et al. *JAMA Network Open*

eTable 3: Risks and excess burdens of pre-specified new-incident autoimmune diagnoses in infected cases and test-negative control groups during Delta and Omicron BA.1/2 periods, completed full primary vaccination series

**Autoimmune diagnoses:**	Cases with diagnosis N(%)[a]	Infected cases (N)[a]	Controls with diagnosis N(%)[a]	Test-negative controls (N)[a]	Excess burden (weighted, per 1000 persons), 95% CI	Adjusted hazards ratio(aHR),[c] 95% CI
**New-incident autoimmune diagnoses in infected cases and test-negative control groups during Delta period (completed full primary vaccination series)**						
Systemic lupus erythematosus	0(0.00)	84483	30(0.01)	519248	-#	-#
Rheumatoid arthritis	25(0.03)	84311	180(0.03)	518398	-0.07 (-0.26-0.12)	0.81(0.53-1.24)
Sjogren's syndrome	1(0.00)	84504	9(0.00)	519305	-0.01 (-0.05-0.03)	0.63(0.08-5.07)
Systemic sclerosis	1(0.00)	84512	5(0.00)	519362	-#	-#
Dermatomyositis/polymyositis	0(0.00)	84512	18(0.00)	519349	-#	-#
Other connective tissue diseases [b]	1(0.00)	84510	4(0.00)	519360	-#	-#
Vasculitis	1(0.00)	84494	27(0.01)	519241	-0.05 (-0.11-0.02)	0.21(0.03-1.57)
Inflammatory bowel disease	51(0.06)	84242	312(0.06)	517611	-0.02 (-0.28-0.24)	0.97(0.72-1.31)
Spondyloarthropathies	9(0.01)	84480	46(0.01)	519153	-0.00 (-0.10-0.10)	0.99(0.48-2.04)
Psoriasis	27(0.03)	84287	159(0.03)	518335	-0.04 (-0.23-0.15)	0.89(0.59-1.34)
Bullous skin disorders	7(0.01)	84498	38(0.01)	519278	-0.02 (-0.12-0.08)	0.80(0.35-1.80)
Autoimmune thyroid disease	16(0.02)	84413	101(0.02)	518808	-0.01 (-0.15-0.14)	0.97(0.57-1.65)
**New-incident autoimmune diagnoses in infected cases and test-negative control groups during Omicron BA.1/2 period (completed full primary vaccination series)**						
**Autoimmune diagnoses:**	Cases with diagnosis N(%)[a]	Infected cases (N)[a]	Controls with diagnosis N(%)[a]	Test-negative controls (N)[a]	Excess burden (weighted, per 1000 persons), 95% CI	Adjusted hazards ratio(aHR),[c] 95% CI
Systemic lupus erythematosus	12(0.01)	83461	23(0.01)	209346	0.03 (-0.10-0.16)	1.27(0.63-2.56)
Rheumatoid arthritis	33(0.04)	83325	56(0.03)	209069	0.12 (-0.09-0.33)	1.44(0.94-2.22)
Sjogren's syndrome	3(0.00)	83485	5(0.00)	209385	-#	-#
Systemic sclerosis	2(0.00)	83498	0(0.00)	209406	-#	-#
Dermatomyositis/polymyositis	0(0.00)	83493	6(0.00)	209400	-#	-#
Other connective tissue diseases [b]	4(0.00)	83492	4(0.00)	209392	-#	-#
Vasculitis	7(0.01)	83468	3(0.00)	209356	0.07 (0.01-0.14)	5.74(1.48-22.23)
Inflammatory bowel disease	66(0.08)	83186	133(0.06)	208764	0.10 (-0.21-0.41)	1.15(0.85-1.55)
Spondyloarthropathies	7(0.01)	83451	27(0.01)	209342	-0.06 (-0.18-0.06)	0.59(0.25-1.36)
Psoriasis	26(0.03)	83317	60(0.03)	209071	0.00 (-0.20-0.20)	1.01(0.64-1.61)
Bullous skin disorders	15(0.02)	83468	14(0.01)	209362	0.08 (-0.04-0.21)	2.08(0.99-4.35)
Autoimmune thyroid disease	20(0.02)	83392	46(0.02)	209182	0.01 (-0.17-0.18)	1.03(0.61-1.75)

HR > 1 denotes higher risk of a respective autoimmune diagnosis amongst infected cases, versus test-negative control group

Excess burden > 0 denotes excess burden in a respective autoimmune diagnosis amongst infected cases, versus test-negative control group

[a] Numbers in each subcohort for each specific autoimmune diagnosis do not add up to the original number of infected cases and test-negative controls because for estimation of risks for each new-incident autoimmune diagnosis, a sub-cohort of individuals without history of the diagnosis in the past 5 years was constructed.

[b] Other connective tissue diseases included: mixed connective tissue disease; Behcet's disease; polymyalgia rheumatica

[c] Each model is overlap weighted and regression adjusted based on demographic characteristics (age, sex, ethnicity), socioeconomic status (housing type), vaccination status (not fully vaccinated, fully vaccinated, fully vaccinated and boosted), and comorbidities

[#] Risks could not be estimated due to too few numbers of new-incident autoimmune diagnosis for that subcategory.

© 2024 Wee LE et al. *JAMA Network Open*

eTable 4: Risks and excess burdens of pre-specified new-incident autoimmune diagnoses in infected cases and test-negative control groups during Delta and Omicron BA.1/2 periods, boosted

**New-incident autoimmune diagnoses in infected cases and test-negative control groups during Delta period (boosted)**						
**Autoimmune diagnoses:**	Cases with diagnosis N(%)[a]	Infected cases (N)[a]	Controls with diagnosis N(%)[a]	Test-negative controls (N)[a]	Excess burden (weighted, per 1000 persons), 95% CI	Adjusted hazards ratio(aHR),[c] 95% CI
Systemic lupus erythematosus	1(0.01)	11050	1(0.01)	93209	-#	-#
Rheumatoid arthritis	2(0.02)	11019	2(0.02)	92899	-0.38 (-0.93-0.18)	0.34(0.08-1.39)
Sjogren's syndrome	0(0.00)	11059	0(0.00)	93212	-#	-#
Systemic sclerosis	0(0.00)	11059	0(0.00)	93217	-#	-#
Dermatomyositis/polymyositis	0(0.00)	11057	0(0.00)	93217	-#	-#
Other connective tissue diseases [b]	1(0.01)	11058	1(0.01)	93216	-#	-#
Vasculitis	1(0.01)	11057	1(0.01)	93193	-#	-#
Inflammatory bowel disease	8(0.07)	11003	8(0.07)	92851	-0.09 (-0.87-0.70)	0.89(0.43-1.86)
Spondyloarthropathies	1(0.01)	11054	1(0.01)	93185	-0.06 (-0.37-0.25)	0.61(0.08-4.76)
Psoriasis	2(0.02)	11018	2(0.02)	92958	-0.18 (-0.65-0.29)	0.51(0.12-2.14)
Bullous skin disorders	1(0.01)	11048	1(0.01)	93189	-0.09 (-0.41-0.23)	0.48(0.06-3.76)
Autoimmune thyroid disease	2(0.02)	11046	2(0.02)	93150	-0.03 (-0.43-0.38)	0.88(0.20-3.79)
**New-incident autoimmune diagnoses in infected cases and test-negative control groups during Omicron BA.1/2 period (boosted)**						
**Autoimmune diagnoses:**	Cases with diagnosis N(%)[a]	Infected cases (N)[a]	Controls with diagnosis N(%)[a]	Test-negative controls (N)[a]	Excess burden (weighted, per 1000 persons), 95% CI	Adjusted hazards ratio(aHR),[c] 95% CI
Systemic lupus erythematosus	19(0.01)	280133	32(0.01)	391608	-0.02 (-0.08-0.04)	0.78(0.44-1.38)
Rheumatoid arthritis	115(0.04)	279484	162(0.04)	390798	0.03 (-0.11-0.17)	1.07(0.84-1.37)
Sjogren's syndrome	17(0.01)	280210	13(0.00)	391652	0.03 (-0.02-0.08)	1.91(0.92-3.96)
Systemic sclerosis	7(0.00)	280233	11(0.00)	391686	-0.00 (-0.04-0.03)	0.91(0.35-2.38)
Dermatomyositis/polymyositis	6(0.00)	280228	18(0.00)	391681	-0.02 (-0.06-0.02)	0.49(0.19-1.26)
Other connective tissue diseases [b]	7(0.00)	280227	3(0.00)	391674	0.02 (-0.01-0.04)	3.38(0.87-13.11)
Vasculitis	14(0.00)	280171	22(0.01)	391587	-0.00 (-0.05-0.05)	0.96(0.49-1.88)
Inflammatory bowel disease	183(0.07)	279260	262(0.07)	390350	0.02 (-0.16-0.19)	1.02(0.85-1.24)
Spondyloarthropathies	33(0.01)	280122	45(0.01)	391523	0.00 (-0.07-0.08)	1.01(0.64-1.60)
Psoriasis	89(0.03)	279471	140(0.04)	390789	-0.04 (-0.17-0.09)	0.88(0.68-1.16)
Bullous skin disorders	31(0.01)	280167	32(0.01)	391631	0.02 (-0.05-0.09)	1.26(0.77-2.08)
Autoimmune thyroid disease	83(0.03)	279910	117(0.03)	391323	-0.00 (-0.12-0.12)	0.99(0.75-1.32)

HR > 1 denotes higher risk of a respective autoimmune diagnosis amongst infected cases, versus test-negative control group

Excess burden > 0 denotes excess burden in a respective autoimmune diagnosis amongst infected cases, versus test-negative control group

[a] Numbers in each subcohort for each specific autoimmune diagnosis do not add up to the original number of infected cases and test-negative controls because for estimation of risks for each new-incident autoimmune diagnosis, a sub-cohort of individuals without history of the diagnosis in the past 5 years was constructed.

[b] Other connective tissue diseases included: mixed connective tissue disease; Behcet's disease; polymyalgia rheumatica

[c] Each model is overlap weighted and regression adjusted based on demographic characteristics (age, sex, ethnicity), socioeconomic status (housing type), vaccination status (not fully vaccinated, fully vaccinated, fully vaccinated and boosted), and comorbidities

[#] Risks could not be estimated due to too few numbers of new-incident autoimmune diagnosis for that subcategory.

© 2024 Wee LE et al. *JAMA Network Open*

eTable 5: Risks and excess burdens of pre-specified new-incident autoimmune diagnoses in infected cases and test-negative control groups during Delta and Omicron BA.1/2 periods, using inverse propensity weighting

Autoimmune diagnoses:	Cases with diagnosis N(%)[a]	Infected cases (N) [a]	Controls with diagnosis N(%)[a]	Test-negative controls (N) [a]	Excess burden (weighted, per 1000 persons), 95% CI	Adjusted hazards ratio(aHR),[c] 95% CI
**New-incident autoimmune diagnoses in infected cases and test-negative control groups during Delta period**						
Systemic lupus erythematosus	2(0.00)	104125	47(0.01)	666367	-0.04 (-0.10-0.02)	0.32(0.08-1.35)
Rheumatoid arthritis	34(0.03)	103902	250(0.04)	665098	-0.06 (-0.24-0.11)	0.85(0.59-1.22)
Sjogren's syndrome	1(0.00)	104162	11(0.00)	666463	-#	-#
Systemic sclerosis	1(0.00)	104170	6(0.00)	666535	-#	-#
Dermatomyositis/polymyositis	0(0.00)	104168	28(0.00)	666515	-#	-#
Other connective tissue diseases [b]	2(0.00)	104169	8(0.00)	666531	0.01 (-0.03-0.05)	1.86(0.38-9.00)
Vasculitis	2(0.00)	104144	37(0.01)	666363	-0.04 (-0.10-0.02)	0.33(0.08-1.38)
Inflammatory bowel disease	66(0.06)	103806	422(0.06)	664169	-0.03 (-0.27-0.21)	0.96(0.74-1.25)
Spondyloarthropathies	10(0.01)	104125	64(0.01)	666265	-0.02 (-0.11-0.07)	0.84(0.42-1.66)
Psoriasis	31(0.03)	103883	210(0.03)	665114	-0.07 (-0.24-0.10)	0.82(0.56-1.20)
Bullous skin disorders	11(0.01)	104139	62(0.01)	666401	-0.03 (-0.12-0.07)	0.85(0.44-1.63)
Autoimmune thyroid disease	20(0.02)	104050	142(0.02)	665825	-0.02 (-0.16-0.11)	0.90(0.56-1.44)
**New-incident autoimmune diagnoses in infected cases and test-negative control groups during Omicron BA.1/2 period**						
**Autoimmune diagnoses:**	Cases with diagnosis N(%)[a]	Infected cases (N) [a]	Controls with diagnosis N(%)[a]	Test-negative controls (N) [a]	Excess burden (weighted, per 1000 persons), 95% CI	Adjusted hazards ratio(aHR),[c] 95% CI
Systemic lupus erythematosus	31(0.01)	375722	58(0.01)	619189	0.90(0.58-1.41)	-0.01 (-0.06-0.05)
Rheumatoid arthritis	151(0.04)	374920	221(0.04)	618087	1.15(0.93-1.41)	0.05 (-0.06-0.17)
Sjogren's syndrome	21(0.01)	375833	18(0.00)	619290	1.89(1.00-3.59)	0.03 (-0.01-0.06)
Systemic sclerosis	10(0.00)	375873	12(0.00)	619348	1.26(0.54-2.91)	0.01 (-0.02-0.03)
Dermatomyositis/polymyositis	6(0.00)	375863	24(0.00)	619338	-#	-#
Other connective tissue diseases [b]	11(0.00)	375859	8(0.00)	619320	2.40(0.95-6.06)	0.02 (-0.01-0.05)
Vasculitis	22(0.01)	375772	26(0.00)	619198	1.35(0.76-2.38)	0.02 (-0.03-0.06)
Inflammatory bowel disease	261(0.07)	374520	406(0.07)	617308	1.06(0.91-1.25)	0.05 (-0.10-0.20)
Spondyloarthropathies	44(0.01)	375711	74(0.01)	619113	0.92(0.63-1.35)	-0.01 (-0.07-0.05)
Psoriasis	121(0.03)	374902	203(0.03)	618085	0.94(0.75-1.18)	-0.02 (-0.12-0.09)
Bullous skin disorders	52(0.01)	375762	49(0.01)	619243	1.49(1.01-2.21)	0.05 (-0.01-0.11)
Autoimmune thyroid disease	106(0.03)	375426	170(0.03)	618744	0.99(0.77-1.26)	-0.00 (-0.10-0.09)

HR > 1 denotes higher risk of a respective autoimmune diagnosis amongst infected cases, versus test-negative control group

Excess burden > 0 denotes excess burden in a respective autoimmune diagnosis amongst infected cases, versus test-negative control group

[a] Numbers in each subcohort for each specific autoimmune diagnosis do not add up to the original number of infected cases and test-negative controls because for estimation of risks for each new-incident autoimmune diagnosis, a sub-cohort of individuals without history of the diagnosis in the past 5 years was constructed.

[b] Other connective tissue diseases included: mixed connective tissue disease; Behcet's disease; polymyalgia rheumatica

[c] Each model is inverse propensity weighted and regression adjusted based on demographic characteristics (age, sex, ethnicity), socioeconomic status (housing type), vaccination status (not fully vaccinated, fully vaccinated, fully vaccinated and boosted), and comorbidities

[#] Risks could not be estimated due to too few numbers of new-incident autoimmune diagnosis for that subcategory.

© 2024 Wee LE et al. *JAMA Network Open*

eTable 6: Risks and excess burdens of pre-specified new-incident autoimmune diagnoses in infected cases and test-negative control groups during Delta and Omicron BA.1/2 periods stratified by initial infection severity, using inverse propensity weighting

	Mild cases not requiring hospitalization (N=93,865), [a] versus test-negatives; Delta-predominant transmission		Hospitalized cases (N=10.314),[a] versus test-negatives; Delta-predominant transmission		Mild cases not requiring hospitalization (N=368,385), [a] versus test-negatives; Omicron-predominant transmission		Hospitalized cases (N=7,518),[a] versus test-negatives; Omicron-predominant transmission	
**Autoimmune diagnoses:**	**Adjusted hazards ratio(aHR),[c] 95% CI**	**Excess burden (weighted, per 1000 persons), 95% CI**	**Adjusted hazards ratio(aHR), [c] 95% CI**	**Excess burden (weighted, per 1000 persons), 95% CI**	**Adjusted hazards ratio(aHR), [c] 95% CI**	**Excess burden (weighted, per 1000 persons), 95% CI**	**Adjusted hazards ratio(aHR), [c] 95% CI**	**Excess burden (weighted, per 1000 persons), 95% CI**
Systemic lupus erythematosus	0.19(0.03-1.34)	-0.05 (-0.11-0.01)	1.56(0.24-10.21)	0.06 (-0.25-0.37)	0.86(0.54-1.36)	-0.01 (-0.07-0.04)	3.41(0.97-11.94)	0.27 (-0.25-0.80)
Rheumatoid arthritis	0.84(0.56-1.26)	-0.08 (-0.25-0.10)	0.78(0.30-2.00)	-0.10 (-0.80-0.60)	1.17(0.95-1.45)	0.06 (-0.06-0.17)	1.13(0.46-2.78)	0.09 (-0.72-0.90)
Sjogren's syndrome	-#	-#	-#	-#	1.84(0.95-3.56)	0.02 (-0.01-0.06)	-#	-#
Systemic sclerosis	-#	-#	-#	-#	1.18(0.49-2.84)	0.00 (-0.02-0.03)	3.03(0.30-30.98)	0.10 (-0.19-0.40)
Dermatomyositis/polymyositis	-#	-#	-#	-#	0.42(0.17-1.05)	-0.02 (-0.05-0.01)	-#	-#
Other connective tissue diseases [b]	2.39(0.51-11.27)	0.01 (-0.03-0.05)	-#	-#	2.43(0.95-6.17)	0.02 (-0.01-0.04)	-#	-#
Vasculitis	0.41(0.09-1.77)	-0.03 (-0.10-0.03)	-#	-#	1.22(0.68-2.22)	0.01 (-0.03-0.05)	6.60(1.23-35.39)	0.23 (-0.18-0.63)
Inflammatory bowel disease	0.85(0.63-1.16)	-0.10 (-0.33-0.13)	1.32(0.75-2.35)	0.40 (-0.77-1.57)	1.01(0.86-1.18)	0.01 (-0.14-0.16)	2.23(1.45-3.44)	1.78 (0.19-3.36)
Spondyloarthropathies	0.71(0.32-1.58)	-0.03 (-0.12-0.06)	2.10(0.62-7.12)	0.19 (-0.31-0.70)	0.82(0.55-1.22)	-0.02 (-0.08-0.04)	2.36(0.97-5.71)	0.45 (-0.29-1.19)
Psoriasis	0.84(0.56-1.25)	-0.06 (-0.23-0.11)	0.67(0.22-2.07)	-0.14 (-0.79-0.51)	0.93(0.74-1.18)	-0.02 (-0.13-0.08)	1.70(0.74-3.90)	0.34 (-0.48-1.15)
Bullous skin disorders	0.25(0.06-1.00)	-0.08 (-0.16--0.00)	2.32(1.06-5.08)	0.48 (-0.33-1.30)	1.17(0.75-1.82)	0.01 (-0.04-0.07)	4.88(2.44-9.76)	1.51 (0.41-2.62)
Autoimmune thyroid disease	0.96(0.59-1.57)	-0.01 (-0.15-0.13)	0.64(0.17-2.49)	-0.12 (-0.67-0.43)	0.97(0.76-1.25)	-0.01 (-0.11-0.09)	1.44(0.57-3.60)	0.23 (-0.54-1.01)

HR > 1 denotes higher risk of a respective autoimmune diagnosis amongst infected cases, versus test-negative control group
Excess burden > 0 denotes excess burden in a respective autoimmune diagnosis amongst infected cases, versus test-negative control group
[a] Infected cases were stratified by severity of initial infection (mild cases managed in ambulatory care alone, and cases requiring hospitalisation). Results for the subgroup of individuals who had severe COVID-19 disease (requiring oxygen, or intensive-care-unit/high-dependency admission) were not presented as risks of individual autoimmune diagnoses could not be estimated due to too few cases; however, there was no increased risk of any autoimmune diagnosis (composite outcome) in severe COVID-19 cases, compared to test-negatives during Delta (aHR=1.40, 95%CI=0.86-2.31) and Omicron (aHR=1.65, 95%CI=1.00-2.71)
[b] Other connective tissue diseases included: mixed connective tissue disease; Behcet's disease; polymyalgia rheumatica
[c] Each model is inverse propensity weighted and regression adjusted based on demographic characteristics (age, sex, ethnicity), socioeconomic status (housing type), vaccination status (not fully vaccinated, fully vaccinated, fully vaccinated and boosted), and comorbidities
[#] Risks could not be estimated due to too few numbers of new-incident autoimmune diagnosis for that subcategory.

© 2024 Wee LE et al. *JAMA Network Open*

eTable 7: Risks and excess burdens of pre-specified new-incident autoimmune diagnoses in infected cases and test-negative control groups during Delta and Omicron BA.1/2 periods stratified by vaccination status, using inverse propensity weighting

	Fully vaccinated cases (N=84,518), [a] versus test-negatives; Delta-predominant transmission		Boosted cases (N=11,059),[a] versus test-negatives; Delta-predominant transmission		Fully vaccinated cases (N=83,506), [a] versus test-negatives; Omicron-predominant transmission		Boosted cases (N=280,254),[a] versus test-negatives; Omicron-predominant transmission	
**Autoimmune diagnoses:**	**Adjusted hazards ratio(aHR),[c] 95% CI**	**Excess burden (weighted, per 1000 persons), 95% CI**	**Adjusted hazards ratio(aHR), [c] 95% CI**	**Excess burden (weighted, per 1000 persons), 95% CI**	**Adjusted hazards ratio(aHR), [c] 95% CI**	**Excess burden (weighted, per 1000 persons), 95% CI**	**Adjusted hazards ratio(aHR), [c] 95% CI**	**Excess burden (weighted, per 1000 persons), 95% CI**
Systemic lupus erythematosus	-#	-#	-#	-#	1.27(0.63-2.57)	0.03 (-0.10-0.16)	0.79(0.44-1.40)	-0.02 (-0.08-0.04)
Rheumatoid arthritis	0.84(0.55-1.29)	-0.07 (-0.26-0.12)	0.34(0.08-1.43)	-0.38 (-0.93-0.18)	1.42(0.92-2.19)	0.12 (-0.09-0.33)	1.08(0.85-1.38)	0.03 (-0.11-0.17)
Sjogren's syndrome	0.66(0.08-5.36)	-0.01 (-0.05-0.03)	-#	-#	-#	-#	1.95(0.94-4.06)	0.03 (-0.02-0.08)
Systemic sclerosis	-#	-#	-#	-#	-#	-#	0.92(0.36-2.39)	-0.00 (-0.04-0.03)
Dermatomyositis/polymyositis	-#	-#	-#	-#	-#	-#	0.50(0.20-1.25)	-0.02 (-0.06-0.02)
Other connective tissue diseases [b]	-#	-#	-#	-#	-#	-#	3.37(0.87-13.09)	0.02 (-0.01-0.04)
Vasculitis	0.22(0.03-1.66)	-0.05 (-0.11-0.02)	-#	-#	5.68(1.48-21.87)	0.07 (-0.01-0.14)	0.97(0.49-1.90)	-0.00 (-0.05-0.05)
Inflammatory bowel disease	0.98(0.72-1.32)	-0.02 (-0.28-0.24)	0.90(0.43-1.87)	-0.09 (-0.87-0.70)	1.14(0.85-1.54)	0.10 (-0.21-0.41)	1.02(0.84-1.23)	0.02 (-0.16-0.19)
Spondyloarthropathies	1.00(0.48-2.08)	-0.00 (-0.10-0.10)	0.62(0.08-4.75)	-0.06 (-0.37-0.25)	0.59(0.26-1.37)	-0.06 (-0.18-0.06)	1.01(0.64-1.59)	0.00 (-0.07-0.08)
Psoriasis	0.89(0.59-1.35)	-0.04 (-0.23-0.15)	0.51(0.12-2.15)	-0.18 (-0.65-0.29)	1.01(0.63-1.60)	0.00 (-0.20-0.20)	0.88(0.67-1.15)	-0.04 (-0.17-0.09)
Bullous skin disorders	0.90(0.39-2.03)	-0.02 (-0.12-0.08)	0.52(0.07-4.04)	-0.09 (-0.41-0.23)	2.07(1.00-4.32)	0.08 (-0.04-0.21)	1.22(0.74-2.01)	0.02 (-0.05-0.09)
Autoimmune thyroid disease	0.98(0.57-1.66)	-0.01 (-0.15-0.14)	0.88(0.20-3.79)	-0.03 (-0.43-0.38)	1.03(0.60-1.75)	0.01 (-0.17-0.18)	1.00(0.75-1.32)	-0.00 (-0.12-0.12)

HR > 1 denotes higher risk of a respective autoimmune diagnosis amongst infected cases, versus test-negative control group
Excess burden > 0 denotes excess burden in a respective autoimmune diagnosis amongst infected cases, versus test-negative control group

[a] Vaccination status was defined as: completed primary vaccination series only (having completed a primary vaccine series of 2 doses of mRNA COVID-19 vaccines, either BNT162b2 or mRNA-1273, at least 8 weeks apart); and having received at least a single booster dose (receiving at least a third mRNA vaccine dose six to nine months after the second dose). Results for the subgroup of individuals who did not complete primary vaccination series (i.e either unvaccinated or partially vaccinated with a single vaccine dose) were not presented as risks of individual autoimmune diagnoses could not be estimated due to too few cases; however, there was no increased risk of any autoimmune diagnosis (composite outcome) in unvaccinated/partially-vaccinated and infected cases, compared to test-negatives, during both Delta (aHR=0.70, 95%CI=0.46-1.06) and Omicron (aHR=1.28, 95%CI=0.82-1.99)

[b] Other connective tissue diseases included: mixed connective tissue disease; Behcet's disease; polymyalgia rheumatica

[c] Each model is inverse propensity weighted and regression adjusted based on demographic characteristics (age, sex, ethnicity), socioeconomic status (housing type), vaccination status (not fully vaccinated, fully vaccinated, fully vaccinated and boosted), and comorbidities

[#] Risks could not be estimated due to too few numbers of new-incident autoimmune diagnosis for that subcategory.

© 2024 Wee LE et al. *JAMA Network Open*

eTable 8: Risks and excess burdens of pre-specified new-incident autoimmune diagnoses in infected cases and test-negative control groups during Delta and Omicron BA.1/2 periods (male subgroup)

**New-incident autoimmune diagnoses in infected cases and test-negative control groups during Delta period**						
**Autoimmune diagnoses:**	Cases with diagnosis N(%)[a]	Infected cases (N)[a]	Controls with diagnosis N(%)[a]	Test-negative controls (N)[a]	Excess burden (weighted, per 1000 persons), 95% CI	Adjusted hazards ratio(aHR),[c] 95% CI
Systemic lupus erythematosus	0(0.00)	58823	6(0.00)	313728	-#	-#
Rheumatoid arthritis	10(0.02)	58757	64(0.02)	313424	-0.07 (-0.25-0.11)	0.69(0.35-1.36)
Sjogren's syndrome	0(0.00)	58831	2(0.00)	313745	-#	-#
Systemic sclerosis	1(0.00)	58835	0(0.00)	313748	-#	-#
Dermatomyositis/polymyositis	0(0.00)	58831	11(0.00)	313725	-#	-#
Other connective tissue diseases [b]	0(0.00)	58832	3(0.00)	313741	-#	-#
Vasculitis	2(0.00)	58821	19(0.01)	313669	-0.03 (-0.12-0.06)	0.56(0.13-2.42)
Inflammatory bowel disease	40(0.07)	58628	190(0.06)	312607	0.05 (-0.27-0.38)	1.09(0.77-1.54)
Spondyloarthropathies	8(0.01)	58810	35(0.01)	313586	0.01 (-0.13-0.15)	1.07(0.49-2.34)
Psoriasis	16(0.03)	58633	115(0.04)	312853	-0.15 (-0.38-0.09)	0.65(0.38-1.10)
Bullous skin disorders	6(0.01)	58814	23(0.01)	313669	-0.01 (-0.13-0.12)	0.92(0.37-2.26)
Autoimmune thyroid disease	4(0.01)	58800	24(0.01)	313579	-0.02 (-0.13-0.09)	0.75(0.26-2.17)
**New-incident autoimmune diagnoses in infected cases and test-negative control groups during Omicron BA.1/2 period**						
**Autoimmune diagnoses:**	Cases with diagnosis N(%)[a]	Infected cases (N)[a]	Controls with diagnosis N(%)[a]	Test-negative controls (N)[a]	Excess burden (weighted, per 1000 persons), 95% CI	Adjusted hazards ratio(aHR),[c] 95% CI
Systemic lupus erythematosus	3(0.00)	184454	10(0.00)	293851	-0.02 (-0.06-0.02)	0.48(0.13-1.76)
Rheumatoid arthritis	29(0.02)	184274	54(0.02)	293601	-0.03 (-0.14-0.08)	0.84(0.53-1.33)
Sjogren's syndrome	0(0.00)	184471	2(0.00)	293860	-#	-#
Systemic sclerosis	4(0.00)	184477	2(0.00)	293868	-#	-#
Dermatomyositis/polymyositis	4(0.00)	184471	5(0.00)	293857	-#	-#
Other connective tissue diseases [b]	3(0.00)	184471	2(0.00)	293854	-#	-#
Vasculitis	13(0.01)	184433	7(0.00)	293802	0.05 (-0.01-0.11)	2.91(1.14-7.44)
Inflammatory bowel disease	144(0.08)	183789	190(0.06)	292918	0.15 (-0.08-0.37)	1.23(0.99-1.53)
Spondyloarthropathies	28(0.02)	184373	41(0.01)	293729	0.01 (-0.09-0.11)	1.05(0.65-1.72)
Psoriasis	73(0.04)	183865	134(0.05)	293060	-0.07 (-0.24-0.11)	0.86(0.64-1.14)
Bullous skin disorders	26(0.01)	184405	19(0.01)	293804	0.06 (-0.03-0.14)	1.74(0.95-3.17)
Autoimmune thyroid disease	30(0.02)	184363	42(0.01)	293738	-0.00 (-0.11-0.10)	0.99(0.61-1.58)

HR > 1 denotes higher risk of a respective autoimmune diagnosis amongst infected cases, versus test-negative control group

Excess burden > 0 denotes excess burden in a respective autoimmune diagnosis amongst infected cases, versus test-negative control group

[a] Numbers in each subcohort for each specific autoimmune diagnosis do not add up to the original number of infected cases and test-negative controls because for estimation of risks for each new-incident autoimmune diagnosis, a sub-cohort of individuals without history of the diagnosis in the past 5 years was constructed.

[b] Other connective tissue diseases included: mixed connective tissue disease; Behcet's disease; polymyalgia rheumatica

[c] Each model is overlap weighted and regression adjusted based on demographic characteristics (age, sex, ethnicity), socioeconomic status (housing type), vaccination status (not fully vaccinated, fully vaccinated, fully vaccinated and boosted), and comorbidities

[#] Risks could not be estimated due to too few numbers of new-incident autoimmune diagnosis for that subcategory.

© 2024 Wee LE et al. *JAMA Network Open*

eTable 9: Risks and excess burdens of pre-specified new-incident autoimmune diagnoses in infected cases and test-negative control groups during Delta and Omicron BA.1/2 periods (female subgroup)

New-incident autoimmune diagnoses in infected cases and test-negative control groups during Delta period

**Autoimmune diagnoses:**	Cases with diagnosis N(%)[a]	Infected cases (N)[a]	Controls with diagnosis N(%)[a]	Test-negative controls (N)[a]	Excess burden (weighted, per 1000 persons), 95% CI	Adjusted hazards ratio(aHR),[c] 95% CI
Systemic lupus erythematosus	41(0.01)	352639	2(0.00)	45302	-0.07 (-0.20-0.05)	0.38(0.09-1.56)
Rheumatoid arthritis	186(0.05)	351674	24(0.05)	45145	-0.05 (-0.38-0.27)	0.91(0.59-1.39)
Sjogren's syndrome	9(0.00)	352718	1(0.00)	45331	-0.01 (-0.08-0.06)	0.79(0.10-6.38)
Systemic sclerosis	6(0.00)	352787	0(0.00)	45335	-#	-#
Dermatomyositis/polymyositis	17(0.00)	352790	0(0.00)	45337	-#	-#
Other connective tissue diseases [b]	5(0.00)	352790	2(0.00)	45337	-#	-#
Vasculitis	18(0.01)	352694	0(0.00)	45323	-#	-#
Inflammatory bowel disease	232(0.07)	351562	26(0.06)	45178	-0.13 (-0.48-0.22)	0.81(0.54-1.22)
Spondyloarthropathies	29(0.01)	352679	2(0.00)	45315	-0.05 (-0.16-0.07)	0.47(0.11-1.98)
Psoriasis	95(0.03)	352261	15(0.03)	45250	0.03 (-0.22-0.27)	1.09(0.63-1.88)
Bullous skin disorders	39(0.01)	352732	5(0.01)	45325	-0.05 (-0.20-0.11)	0.69(0.27-1.76)
Autoimmune thyroid disease	118(0.03)	352246	16(0.04)	45250	-0.03 (-0.30-0.24)	0.93(0.55-1.57)

New-incident autoimmune diagnoses in infected cases and test-negative control groups during Omicron BA.1/2 period

**Autoimmune diagnoses:**	Cases with diagnosis N(%)[a]	Infected cases (N)[a]	Controls with diagnosis N(%)[a]	Test-negative controls (N)[a]	Excess burden (weighted, per 1000 persons), 95% CI	Adjusted hazards ratio(aHR),[c] 95% CI
Systemic lupus erythematosus	28(0.01)	191268	28(0.01)	325338	0.00 (-0.10-0.10)	1.00(0.62-1.61)
Rheumatoid arthritis	122(0.06)	190646	122(0.06)	324486	0.13 (-0.07-0.33)	1.25(0.99-1.58)
Sjogren's syndrome	21(0.01)	191362	21(0.01)	325430	0.06 (-0.02-0.13)	2.13(1.10-4.14)
Systemic sclerosis	6(0.00)	191396	6(0.00)	325480	-0.00 (-0.05-0.04)	0.88(0.32-2.43)
Dermatomyositis/polymyositis	2(0.00)	191392	2(0.00)	325481	-0.05 (-0.10--0.00)	0.17(0.04-0.74)
Other connective tissue diseases [b]	8(0.00)	191388	8(0.00)	325466	0.03 (-0.02-0.07)	2.45(0.83-7.21)
Vasculitis	9(0.00)	191339	9(0.00)	325396	-0.02 (-0.08-0.04)	0.75(0.33-1.66)
Inflammatory bowel disease	117(0.06)	190731	117(0.06)	324390	-0.05 (-0.25-0.16)	0.93(0.74-1.17)
Spondyloarthropathies	16(0.01)	191338	16(0.01)	325384	-0.02 (-0.10-0.05)	0.77(0.42-1.40)
Psoriasis	48(0.03)	191037	48(0.03)	325025	0.02 (-0.10-0.15)	1.11(0.76-1.62)
Bullous skin disorders	26(0.01)	191357	26(0.01)	325439	0.04 (-0.05-0.13)	1.43(0.84-2.44)
Autoimmune thyroid disease	76(0.04)	191063	76(0.04)	325006	-0.01 (-0.17-0.16)	0.99(0.74-1.31)

HR > 1 denotes higher risk of a respective autoimmune diagnosis amongst infected cases, versus test-negative control group

Excess burden > 0 denotes excess burden in a respective autoimmune diagnosis amongst infected cases, versus test-negative control group

[a] Numbers in each subcohort for each specific autoimmune diagnosis do not add up to the original number of infected cases and test-negative controls because for estimation of risks for each new-incident autoimmune diagnosis, a sub-cohort of individuals without history of the diagnosis in the past 5 years was constructed.

[b] Other connective tissue diseases included: mixed connective tissue disease; Behcet's disease; polymyalgia rheumatica

[c] Each model is overlap weighted and regression adjusted based on demographic characteristics (age, sex, ethnicity), socioeconomic status (housing type), vaccination status (not fully vaccinated, fully vaccinated, fully vaccinated and boosted), and comorbidities

[#] Risks could not be estimated due to too few numbers of new-incident autoimmune diagnosis for that subcategory.

© 2024 Wee LE et al. *JAMA Network Open*

eTable 10: Risks and excess burdens of pre-specified new-incident autoimmune diagnoses in infected cases and test-negative control groups during Delta and Omicron BA.1/2 periods (age 18-64 years subgroup)

**Autoimmune diagnoses:**	Cases with diagnosis N(%)[a]	Infected cases (N)[a]	Controls with diagnosis N(%)[a]	Test-negative controls (N)[a]	Excess burden (weighted, per 1000 persons), 95% CI	Adjusted hazards ratio(aHR),[c] 95% CI
**New-incident autoimmune diagnoses in infected cases and test-negative control groups during Delta period**						
Systemic lupus erythematosus	1(0.00)	82481	39(0.01)	537659	-0.05 (-0.12-0.01)	0.19(0.03-1.38)
Rheumatoid arthritis	21(0.03)	82372	124(0.02)	537107	0.01 (-0.16-0.18)	1.04(0.65-1.66)
Sjogren's syndrome	0(0.00)	82514	5(0.00)	537764	-#	-#
Systemic sclerosis	1(0.00)	82518	3(0.00)	537820	-#	-#
Dermatomyositis/polymyositis	0(0.00)	82515	15(0.00)	537807	-#	-#
Other connective tissue diseases [b]	0(0.00)	82517	4(0.00)	537816	-#	-#
Vasculitis	1(0.00)	82502	24(0.00)	537691	-0.04 (-0.10-0.02)	0.23(0.03-1.73)
Inflammatory bowel disease	34(0.04)	82310	274(0.05)	536265	-0.08 (-0.30-0.14)	0.84(0.58-1.20)
Spondyloarthropathies	6(0.01)	82485	46(0.01)	537617	-0.03 (-0.13-0.06)	0.68(0.28-1.61)
Psoriasis	23(0.03)	82329	136(0.03)	536887	-0.02 (-0.19-0.16)	0.94(0.60-1.47)
Bullous skin disorders	1(0.00)	82517	17(0.00)	537804	-0.03 (-0.09-0.02)	0.25(0.03-1.90)
Autoimmune thyroid disease	17(0.02)	82429	94(0.02)	537270	0.02 (-0.13-0.17)	1.12(0.66-1.89)
**New-incident autoimmune diagnoses in infected cases and test-negative control groups during Omicron BA.1/2 period**						
**Autoimmune diagnoses:**	Cases with diagnosis N(%)[a]	Infected cases (N)[a]	Controls with diagnosis N(%)[a]	Test-negative controls (N)[a]	Excess burden (weighted, per 1000 persons), 95% CI	Adjusted hazards ratio(aHR),[c] 95% CI
Systemic lupus erythematosus	17(0.01)	304245	17(0.01)	509628	-0.03 (-0.09-0.02)	0.63(0.36-1.11)
Rheumatoid arthritis	81(0.03)	303882	81(0.03)	509134	0.05 (-0.05-0.15)	1.21(0.91-1.61)
Sjogren's syndrome	12(0.00)	304358	12(0.00)	509720	0.02 (-0.01-0.05)	2.26(0.91-5.64)
Systemic sclerosis	3(0.00)	304390	3(0.00)	509768	-0.01 (-0.03-0.02)	0.56(0.14-2.17)
Dermatomyositis/polymyositis	3(0.00)	304377	3(0.00)	509764	-0.01 (-0.04-0.01)	0.47(0.13-1.68)
Other connective tissue diseases [b]	8(0.00)	304386	8(0.00)	509752	0.02 (-0.00-0.05)	4.78(1.24-18.35)
Vasculitis	12(0.00)	304324	12(0.00)	509650	0.01 (-0.02-0.05)	1.48(0.68-3.23)
Inflammatory bowel disease	149(0.05)	303532	149(0.05)	508396	0.00 (-0.14-0.15)	1.01(0.82-1.24)
Spondyloarthropathies	25(0.01)	304270	25(0.01)	509588	-0.01 (-0.07-0.05)	0.90(0.55-1.47)
Psoriasis	86(0.03)	303758	86(0.03)	508925	-0.00 (-0.11-0.10)	0.98(0.75-1.29)
Bullous skin disorders	8(0.00)	304370	8(0.00)	509756	-0.00 (-0.04-0.03)	0.87(0.36-2.08)
Autoimmune thyroid disease	70(0.02)	304054	70(0.02)	509327	-0.01 (-0.11-0.09)	0.95(0.70-1.28)

HR > 1 denotes higher risk of a respective autoimmune diagnosis amongst infected cases, versus test-negative control group

Excess burden > 0 denotes excess burden in a respective autoimmune diagnosis amongst infected cases, versus test-negative control group

[a] Numbers in each subcohort for each specific autoimmune diagnosis do not add up to the original number of infected cases and test-negative controls because for estimation of risks for each new-incident autoimmune diagnosis, a sub-cohort of individuals without history of the diagnosis in the past 5 years was constructed.

[b] Other connective tissue diseases included: mixed connective tissue disease; Behcet's disease; polymyalgia rheumatica

[c] Each model is overlap weighted and regression adjusted based on demographic characteristics (age, sex, ethnicity), socioeconomic status (housing type), vaccination status (not fully vaccinated, fully vaccinated, fully vaccinated and boosted), and comorbidities

[#] Risks could not be estimated due to too few numbers of new-incident autoimmune diagnosis for that subcategory.

© 2024 Wee LE et al. *JAMA Network Open*

eTable 11: Risks and excess burdens of pre-specified new-incident autoimmune diagnoses in infected cases and test-negative control groups during Delta and Omicron BA.1/2 periods (age≥65 years subgroup)

New-incident autoimmune diagnoses in infected cases and test-negative control groups during Delta period

**Autoimmune diagnoses:**	Cases with diagnosis N(%)[a]	Infected cases (N) [a]	Controls with diagnosis N(%)[a]	Test-negative controls (N) [a]	Excess burden (weighted, per 1000 persons), 95% CI	Adjusted hazards ratio(aHR),[c] 95% CI
Systemic lupus erythematosus	1(0.00)	21644	8(0.01)	128708	-#	-#
Rheumatoid arthritis	13(0.06)	21530	126(0.10)	127991	-0.32 (-0.89-0.26)	0.65(0.37-1.17)
Sjogren's syndrome	1(0.00)	21648	6(0.00)	128699	-#	-#
Systemic sclerosis	0(0.00)	21652	3(0.00)	128715	-#	-#
Dermatomyositis/polymyositis	0(0.00)	21653	13(0.01)	128708	-#	-#
Other connective tissue diseases [b]	2(0.01)	21652	4(0.00)	128715	-#	-#
Vasculitis	1(0.00)	21642	13(0.01)	128672	-0.05 (-0.23-0.13)	0.50(0.06-3.83)
Inflammatory bowel disease	32(0.15)	21496	148(0.12)	127904	0.23 (-0.54-0.99)	1.18(0.80-1.75)
Spondyloarthropathies	4(0.02)	21640	18(0.01)	128648	0.03 (-0.24-0.29)	1.17(0.39-3.53)
Psoriasis	8(0.04)	21554	74(0.06)	128227	-0.24 (-0.70-0.21)	0.60(0.29-1.26)
Bullous skin disorders	10(0.05)	21622	45(0.03)	128597	0.04 (-0.38-0.47)	1.11(0.55-2.21)
Autoimmune thyroid disease	3(0.01)	21621	48(0.04)	128555	-0.23 (-0.56-0.11)	0.39(0.12-1.26)

New-incident autoimmune diagnoses in infected cases and test-negative control groups during Omicron BA.1/2 period

**Autoimmune diagnoses:**	Cases with diagnosis N(%)[a]	Infected cases (N) [a]	Controls with diagnosis N(%)[a]	Test-negative controls (N) [a]	Excess burden (weighted, per 1000 persons), 95% CI	Adjusted hazards ratio(aHR),[c] 95% CI
Systemic lupus erythematosus	14(0.02)	71477	15(0.01)	109561	0.09 (-0.09-0.27)	1.69(0.81-3.52)
Rheumatoid arthritis	70(0.10)	71038	106(0.10)	108953	0.11 (-0.32-0.53)	1.12(0.82-1.51)
Sjogren's syndrome	9(0.01)	71475	10(0.01)	109570	0.04 (-0.10-0.18)	1.52(0.61-3.81)
Systemic sclerosis	7(0.01)	71483	5(0.00)	109580	0.06 (-0.06-0.17)	2.32(0.73-7.33)
Dermatomyositis/polymyositis	3(0.00)	71486	12(0.01)	109574	-0.07 (-0.18-0.05)	0.37(0.10-1.32)
Other connective tissue diseases [b]	3(0.00)	71473	5(0.00)	109568	-#	-#
Vasculitis	10(0.01)	71448	12(0.01)	109548	0.03 (-0.12-0.18)	1.27(0.54-2.98)
Inflammatory bowel disease	112(0.16)	70988	141(0.13)	108912	0.21 (-0.30-0.73)	1.16(0.90-1.50)
Spondyloarthropathies	19(0.03)	71441	28(0.03)	109525	-0.01 (-0.23-0.21)	0.96(0.53-1.73)
Psoriasis	35(0.05)	71144	61(0.06)	109160	-0.06 (-0.37-0.25)	0.88(0.58-1.35)
Bullous skin disorders	44(0.06)	71392	34(0.03)	109487	0.27 (-0.02-0.57)	1.80(1.14-2.83)
Autoimmune thyroid disease	36(0.05)	71372	53(0.05)	109417	0.03 (-0.27-0.33)	1.06(0.69-1.63)

HR > 1 denotes higher risk of a respective autoimmune diagnosis amongst infected cases, versus test-negative control group

Excess burden > 0 denotes excess burden in a respective autoimmune diagnosis amongst infected cases, versus test-negative control group

[a] Numbers in each subcohort for each specific autoimmune diagnosis do not add up to the original number of infected cases and test-negative controls because for estimation of risks for each new-incident autoimmune diagnosis, a sub-cohort of individuals without history of the diagnosis in the past 5 years was constructed.

[b] Other connective tissue diseases included: mixed connective tissue disease; Behcet's disease; polymyalgia rheumatica

[c] Each model is overlap weighted and regression adjusted based on demographic characteristics (age, sex, ethnicity), socioeconomic status (housing type), vaccination status (not fully vaccinated, fully vaccinated, fully vaccinated and boosted), and comorbidities

[#] Risks could not be estimated due to too few numbers of new-incident autoimmune diagnosis for that subcategory.

© 2024 Wee LE et al. *JAMA Network Open*

eTable 12: Risks and excess burdens of pre-specified new-incident autoimmune diagnoses in infected cases and test-negative control groups during Delta and Omicron BA.1/2 periods (Chinese subgroup)

New-incident autoimmune diagnoses in infected cases and test-negative control groups during Delta period

**Autoimmune diagnoses:**	Cases with diagnosis N(%)[a]	Infected cases (N) [a]	Controls with diagnosis N(%)[a]	Test-negative controls (N) [a]	Excess burden (weighted, per 1000 persons), 95% CI	Adjusted hazards ratio(aHR),[c] 95% CI
Systemic lupus erythematosus	2(0.00)	71420	36(0.01)	491571	-0.04 (-0.12-0.04)	0.42(0.10-1.77)
Rheumatoid arthritis	22(0.03)	71262	194(0.04)	490635	-0.10 (-0.32-0.11)	0.75(0.48-1.16)
Sjogren's syndrome	1(0.00)	71439	9(0.00)	491633	-0.01 (-0.05-0.04)	0.70(0.09-5.70)
Systemic sclerosis	0(0.00)	71446	6(0.00)	491698	-#	-#
Dermatomyositis/polymyositis	0(0.00)	71444	22(0.00)	491681	-#	-#
Other connective tissue diseases [b]	0(0.00)	71444	8(0.00)	491696	-#	-#
Vasculitis	2(0.00)	71429	27(0.01)	491566	-0.03 (-0.11-0.04)	0.46(0.11-1.93)
Inflammatory bowel disease	47(0.07)	71182	338(0.07)	489941	-0.09 (-0.38-0.21)	0.88(0.65-1.20)
Spondyloarthropathies	5(0.01)	71415	49(0.01)	491487	-0.04 (-0.14-0.07)	0.66(0.26-1.68)
Psoriasis	18(0.03)	71265	137(0.03)	490752	-0.07 (-0.25-0.12)	0.79(0.48-1.30)
Bullous skin disorders	7(0.01)	71423	50(0.01)	491596	-0.05 (-0.16-0.07)	0.67(0.30-1.48)
Autoimmune thyroid disease	15(0.02)	71368	109(0.02)	491168	-0.02 (-0.18-0.15)	0.93(0.54-1.60)

New-incident autoimmune diagnoses in infected cases and test-negative control groups during Omicron BA.1/2 period

**Autoimmune diagnoses:**	Cases with diagnosis N(%)[a]	Infected cases (N) [a]	Controls with diagnosis N(%)[a]	Test-negative controls (N) [a]	Excess burden (weighted, per 1000 persons), 95% CI	Adjusted hazards ratio(aHR),[c] 95% CI
Systemic lupus erythematosus	27(0.01)	272817	41(0.01)	455324	0.00 (-0.12-0.12)	1.12(0.68-1.83)
Rheumatoid arthritis	106(0.04)	272272	163(0.04)	454522	0.00 (-0.12-0.12)	1.07(0.84-1.38)
Sjogren's syndrome	13(0.00)	272887	16(0.00)	455394	0.00 (-0.12-0.12)	1.42(0.68-2.99)
Systemic sclerosis	9(0.00)	272922	9(0.00)	455443	0.00 (-0.12-0.12)	1.53(0.60-3.88)
Dermatomyositis/polymyositis	6(0.00)	272912	21(0.00)	455433	0.00 (-0.12-0.12)	0.45(0.18-1.13)
Other connective tissue diseases [b]	10(0.00)	272910	7(0.00)	455419	0.00 (-0.12-0.12)	2.64(0.99-7.03)
Vasculitis	17(0.01)	272847	20(0.00)	455327	0.00 (-0.12-0.12)	1.37(0.71-2.64)
Inflammatory bowel disease	202(0.07)	271963	325(0.07)	453971	0.00 (-0.12-0.12)	1.05(0.88-1.25)
Spondyloarthropathies	36(0.01)	272805	57(0.01)	455266	0.00 (-0.12-0.12)	1.02(0.67-1.56)
Psoriasis	78(0.03)	272267	132(0.03)	454597	0.00 (-0.12-0.12)	0.95(0.71-1.26)
Bullous skin disorders	38(0.01)	272839	42(0.01)	455372	0.00 (-0.12-0.12)	1.41(0.90-2.20)
Autoimmune thyroid disease	82(0.03)	272611	133(0.03)	454994	0.00 (-0.12-0.12)	1.00(0.76-1.32)

HR > 1 denotes higher risk of a respective autoimmune diagnosis amongst infected cases, versus test-negative control group

Excess burden > 0 denotes excess burden in a respective autoimmune diagnosis amongst infected cases, versus test-negative control group

[a] Numbers in each subcohort for each specific autoimmune diagnosis do not add up to the original number of infected cases and test-negative controls because for estimation of risks for each new-incident autoimmune diagnosis, a sub-cohort of individuals without history of the diagnosis in the past 5 years was constructed.

[b] Other connective tissue diseases included: mixed connective tissue disease; Behcet's disease; polymyalgia rheumatica

[c] Each model is overlap weighted and regression adjusted based on demographic characteristics (age, sex, ethnicity), socioeconomic status (housing type), vaccination status (not fully vaccinated, fully vaccinated, fully vaccinated and boosted), and comorbidities

[#] Risks could not be estimated due to too few numbers of new-incident autoimmune diagnosis for that subcategory.

© 2024 Wee LE et al. *JAMA Network Open*

eTable 13: Risks and excess burdens of pre-specified new-incident autoimmune diagnoses in infected cases and test-negative control groups during Delta and Omicron BA.1/2 periods (Malay subgroup)

Autoimmune diagnoses:	Cases with diagnosis N(%)[a]	Infected cases (N)[a]	Controls with diagnosis N(%)[a]	Test-negative controls (N)[a]	Excess burden (weighted, per 1000 persons), 95% CI	Adjusted hazards ratio(aHR),[c] 95% CI
**New-incident autoimmune diagnoses in infected cases and test-negative control groups during Delta period**						
Systemic lupus erythematosus	0(0.00)	18303	4(0.00)	84852	-#	-#
Rheumatoid arthritis	5(0.03)	18289	23(0.03)	84761	-0.01 (-0.39-0.37)	0.96(0.36-2.56)
Sjogren's syndrome	0(0.00)	18318	2(0.00)	84875	-#	-#
Systemic sclerosis	1(0.01)	18317	0(0.00)	84876	-#	-#
Dermatomyositis/polymyositis	0(0.00)	18318	3(0.00)	84874	-#	-#
Other connective tissue diseases [b]	1(0.01)	18320	0(0.00)	84876	-#	-#
Vasculitis	0(0.00)	18314	2(0.00)	84855	-#	-#
Inflammatory bowel disease	6(0.03)	18279	24(0.03)	84686	-0.03 (-0.43-0.37)	0.91(0.36-2.27)
Spondyloarthropathies	4(0.02)	18313	9(0.01)	84850	0.06 (-0.23-0.35)	1.45(0.44-4.76)
Psoriasis	3(0.02)	18261	35(0.04)	84667	-0.27 (-0.67-0.13)	0.39(0.12-1.27)
Bullous skin disorders	2(0.01)	18315	6(0.01)	84859	-#	-#
Autoimmune thyroid disease	4(0.02)	18300	109(0.02)	84772	0.00 (-0.34-0.35)	1.01(0.34-2.99)
**New-incident autoimmune diagnoses in infected cases and test-negative control groups during Omicron BA.1/2 period**						
**Autoimmune diagnoses:**	Cases with diagnosis N(%)[a]	Infected cases (N)[a]	Controls with diagnosis N(%)[a]	Test-negative controls (N)[a]	Excess burden (weighted, per 1000 persons), 95% CI	Adjusted hazards ratio(aHR),[c] 95% CI
Systemic lupus erythematosus	4(0.01)	62095	5(0.01)	77428	-#	-#
Rheumatoid arthritis	18(0.03)	62000	17(0.02)	77349	0.11 (-0.14-0.35)	1.50(0.77-2.94)
Sjogren's syndrome	5(0.01)	62128	2(0.00)	77452	-#	-#
Systemic sclerosis	1(0.00)	62126	1(0.00)	77455	-#	-#
Dermatomyositis/polymyositis	0(0.00)	62124	1(0.00)	77454	-#	-#
Other connective tissue diseases [b]	1(0.00)	62126	0(0.00)	77451	-#	-#
Vasculitis	4(0.01)	62115	2(0.00)	77442	-#	-#
Inflammatory bowel disease	31(0.05)	61925	26(0.03)	77261	0.12 (-0.19-0.43)	1.34(0.79-2.28)
Spondyloarthropathies	2(0.00)	62108	12(0.02)	77429	-0.14 (-0.28-0.01)	0.16(0.04-0.75)
Psoriasis	25(0.04)	61959	34(0.04)	77301	-0.03 (-0.34-0.28)	0.93(0.55-1.57)
Bullous skin disorders	11(0.02)	62107	4(0.01)	77440	0.12 (-0.04-0.28)	3.44(1.06-11.17)
Autoimmune thyroid disease	19(0.03)	62035	20(0.03)	77361	0.04 (-0.22-0.29)	1.14(0.60-2.16)

HR > 1 denotes higher risk of a respective autoimmune diagnosis amongst infected cases, versus test-negative control group

Excess burden > 0 denotes excess burden in a respective autoimmune diagnosis amongst infected cases, versus test-negative control group

[a] Numbers in each subcohort for each specific autoimmune diagnosis do not add up to the original number of infected cases and test-negative controls because for estimation of risks for each new-incident autoimmune diagnosis, a sub-cohort of individuals without history of the diagnosis in the past 5 years was constructed.

[b] Other connective tissue diseases included: mixed connective tissue disease; Behcet's disease; polymyalgia rheumatica

[c] Each model is overlap weighted and regression adjusted based on demographic characteristics (age, sex, ethnicity), socioeconomic status (housing type), vaccination status (not fully vaccinated, fully vaccinated, fully vaccinated and boosted), and comorbidities

[#] Risks could not be estimated due to too few numbers of new-incident autoimmune diagnosis for that subcategory.

© 2024 Wee LE et al. *JAMA Network Open*

eTable 14: Risks and excess burdens of pre-specified new-incident autoimmune diagnoses in infected cases and test-negative control groups during Delta and Omicron BA.1/2 periods (Indian subgroup)

New-incident autoimmune diagnoses in infected cases and test-negative control groups during Delta period

**Autoimmune diagnoses:**	Cases with diagnosis N(%)[a]	Infected cases (N)[a]	Controls with diagnosis N(%)[a]	Test-negative controls (N)[a]	Excess burden (weighted, per 1000 persons), 95% CI	Adjusted hazards ratio(aHR),[c] 95% CI
Systemic lupus erythematosus	0(0.00)	11669	6(0.01)	67690	-#	-#
Rheumatoid arthritis	6(0.05)	11618	29(0.04)	67471	0.05 (-0.56-0.65)	1.11(0.45-2.72)
Sjogren's syndrome	0(0.00)	11670	0(0.00)	67701	-#	-#
Systemic sclerosis	0(0.00)	11671	0(0.00)	67702	-#	-#
Dermatomyositis/polymyositis	0(0.00)	11670	3(0.00)	67702	-#	-#
Other connective tissue diseases [b]	1(0.01)	11669	0(0.00)	67703	-#	-#
Vasculitis	0(0.00)	11666	5(0.01)	67687	-#	-#
Inflammatory bowel disease	12(0.10)	11615	48(0.07)	67378	0.30 (-0.53-1.13)	1.42(0.74-2.73)
Spondyloarthropathies	1(0.01)	11663	4(0.01)	67678	-#	-#
Psoriasis	9(0.08)	11622	34(0.05)	67467	0.17 (-0.57-0.90)	1.28(0.60-2.73)
Bullous skin disorders	2(0.02)	11665	5(0.01)	67689	-#	-#
Autoimmune thyroid disease	1(0.01)	11652	10(0.01)	67637	-0.08 (-0.39-0.23)	0.49(0.06-3.96)

New-incident autoimmune diagnoses in infected cases and test-negative control groups during Omicron BA.1/2 period

**Autoimmune diagnoses:**	Cases with diagnosis N(%)[a]	Infected cases (N)[a]	Controls with diagnosis N(%)[a]	Test-negative controls (N)[a]	Excess burden (weighted, per 1000 persons), 95% CI	Adjusted hazards ratio(aHR),[c] 95% CI
Systemic lupus erythematosus	0(0.00)	30398	7(0.01)	64325	-#	-#
Rheumatoid arthritis	21(0.07)	30252	35(0.05)	64128	0.12 (-0.38-0.62)	1.20(0.69-2.07)
Sjogren's syndrome	2(0.01)	30401	0(0.00)	64333	-#	-#
Systemic sclerosis	0(0.00)	30411	2(0.00)	64335	-#	-#
Dermatomyositis/polymyositis	0(0.00)	30410	2(0.00)	64336	-#	-#
Other connective tissue diseases [b]	0(0.00)	30407	0(0.00)	64337	-#	-#
Vasculitis	1(0.00)	30396	2(0.00)	64320	-#	-#
Inflammatory bowel disease	25(0.08)	30243	43(0.07)	64054	0.10 (-0.44-0.63)	1.14(0.68-1.91)
Spondyloarthropathies	5(0.02)	30389	4(0.01)	64315	-#	-#
Psoriasis	15(0.05)	30274	31(0.05)	64100	-0.06 (-0.50-0.38)	0.89(0.48-1.67)
Bullous skin disorders	2(0.01)	30400	2(0.00)	64318	-#	-#
Autoimmune thyroid disease	4(0.01)	30375	14(0.02)	64282	-0.10 (-0.37-0.17)	0.58(0.19-1.81)

HR > 1 denotes higher risk of a respective autoimmune diagnosis amongst infected cases, versus test-negative control group

Excess burden > 0 denotes excess burden in a respective autoimmune diagnosis amongst infected cases, versus test-negative control group

[a] Numbers in each subcohort for each specific autoimmune diagnosis do not add up to the original number of infected cases and test-negative controls because for estimation of risks for each new-incident autoimmune diagnosis, a sub-cohort of individuals without history of the diagnosis in the past 5 years was constructed.

[b] Other connective tissue diseases included: mixed connective tissue disease; Behcet's disease; polymyalgia rheumatica

[c] Each model is overlap weighted and regression adjusted based on demographic characteristics (age, sex, ethnicity), socioeconomic status (housing type), vaccination status (not fully vaccinated, fully vaccinated, fully vaccinated and boosted), and comorbidities

[#] Risks could not be estimated due to too few numbers of new-incident autoimmune diagnosis for that subcategory.

© 2024 Wee LE et al. *JAMA Network Open*

eTable 15: Risks and excess burdens of pre-specified negative outcome controls in infected cases vs test-negative control groups during Delta and Omicron BA.1/2 periods

	Controls [a]	Controls with outcome (%)[a]	Cases [a]	Cases with outcome (%)[a]	Adjusted hazards ratio(aHR),[b] 95% CI	Excess burden (weighted, per 1000 persons), 95% CI
**Delta period**						
All negative outcome controls (composite)	665426	338(0.05)	103932	48(0.05)	0.73(0.54-1.00)	-0.16 (-0.37-0.05)
B-cell lymphoma	666192	86(0.01)	104081	16(0.02)	0.94(0.55-1.62)	-0.01 (-0.12-0.11)
Hodgkins lymphoma	666504	14(0.00)	104154	3(0.00)	1.25(0.35-4.43)	0.01 (-0.04-0.05)
Malignancy of the tongue	666499	24(0.00)	104162	6(0.01)	1.32(0.53-3.28)	0.01 (-0.05-0.08)
**Omicron period**						
All negative outcome controls (composite)	618296	296(0.05)	375193	168(0.04)	0.87(0.72-1.05)	-0.07 (-0.19-0.06)
B-cell lymphoma	619070	73(0.01)	375637	59(0.02)	1.16(0.82-1.65)	0.02 (-0.05-0.09)
Hodgkins lymphoma	619292	14(0.00)	375852	9(0.00)	1.10(0.47-2.58)	0.00 (-0.03-0.03)
Malignancy of the tongue	619300	29(0.00)	375852	10(0.00)	0.52(0.25-1.07)	-0.02 (-0.06-0.01)

Hazards ratio (HR) and excess burdens of pre-specified negative outcome controls in the infected and test-negative control groups. 95% confidence intervals are reported in parentheses.
HR > 1 denotes higher risk of a respective composite/individual negative outcomes in the infected versus test-negative control group
Excess burdens > 0 denotes excess burden in a respective composite/individual new negative outcome in the infected versus test-negative control group
[a] Numbers in each subcohort for each specific diagnosis do not add up to the original number of infected cases and test-negative controls because for estimation of risks for each new-incident diagnosis, a sub-cohort of individuals without history of the diagnosis in the past 5 years was constructed.
[b] Each model is overlap weighted and regression adjusted based on demographic characteristics (age, sex, ethnicity), socioeconomic status (housing type), vaccination status (not fully vaccinated, fully vaccinated, fully vaccinated and boosted), and comorbidities

© 2024 Wee LE et al. *JAMA Network Open*

eAppendix: List of ICD-10 codes used for outcomes of interest

	**Outcome**	**ICD 10 Code**	**Description**
**Pre-specified autoimmune outcomes**	Systemic lupus erythematosus	M32	Systemic lupus erythematosus
	Rheumatoid arthritis	M05	Rheumatoid arthritis with rheumatoid factor
		M06	Other rheumatoid arthritis
	Sjogren syndrome	M35.0	Sjogren syndrome
	Systemic sclerosis	M34	Systemic sclerosis
	Dermatomyositis/polymyositis	M33.0	Juvenile dermatomyositis
		M33.1	Other dermatomyositis
		M33.2	Polymyositis
		M33.9	Dermatopolymyositis, unspecified
	Other connective tissue diseases	M35.1	Mixed connective tissue disease, other overlap syndromes
		M35.2	Behcet’s disease
		M35.3	Polymyalgia rheumatica
	Vasculitis	M30.0	Polyarteritis nodosa
		M30.1	Polyarteritis with lung involvement [Churg-Strauss]
		M30.2	Juvenile polyarteritis
		M30.8	Other conditions related to polyarteritis nodosa
		M31.1	Thrombotic microangiopathy
		M31.3	Wegener’s granulomatosis
		M31.4	Aortic arch syndrome [Takayasu]
		M31.5	Giant cell arteritis with polymyalgia rheumatica
		M31.6	Other giant cell arteritis
		M31.7	Microscopic polyangiitis
		M31.8	Other specified necrotizing vasculopathies
		M31.9	Necrotizing vasculopathy, unspecified
		L95	Vasculitis limited to skin, not elsewhere classified
		I77.82	Antineutrophilic cytoplasmic antibody [ANCA] vasculitis
		I79.1	Aortitis in diseases classified elsewhere (isolated aortitis)
		D69.0	Allergic purpura (Henoch-Schonlein purpura)
	Inflammatory bowel disease	K50	Crohn’s disease
		K51	Ulcerative colitis
		K52	Other and unspecified noninfective gastroenteritis and colitis
	Spondyloarthropathies	M45	Ankylosing spondylitis
		M46	Other inflammatory spondylopathies
		M02.30	Reiter’s disease

© 2024 Wee LE et al. *JAMA Network Open*

	M02.1	Postdysenteric arthropathy
Psoriasis	L40.0	Psoriasis vulgaris
	L40.1	Generalised pustular psoriasis
	L40.3	Pustulosis palmaris et plantaris
	L40.4	Guttate psoriasis
	L40.5	Arthropathic psoriasis
	L40.51	Distal interphalangeal psoriatic arthropathy
	L40.52	Psoriatic arthritis mutilans
	L40.52	Psoriatic spondylitis
	L40.54	Psoriatic juvenile arthropathy
	L40.59	Other psoriatic arthropathy
	L40.8	Other psoriasis
	L40.9	Psoriasis, unspecified
Bullous skin disorders	L10	Pemphigus
	L12	Pemphigoid
Autoimmune thyroiditis	E06.3	Autoimmune thyroiditis
	E05.0	Thyrotoxicosis

© 2024 Wee LE et al. *JAMA Network Open*
