# Supplementary material for: Autoimmune Sequelae After Delta or Omicron Variant SARS-CoV-2 Infection in a Highly Vaccinated Cohort
Source: JAMA Netw Open. 2024 Aug 30;7(8):e2430983. doi: 10.1001/jamanetworkopen.2024.30983 (PMC11364997; doi:10.1001/jamanetworkopen.2024.30983)
Supplement: Supplement 2. — Data Sharing Statement [file jamanetwopen-e2430983-s002.pdf]

Data Sharing Statement

Wee. Autoimmune Sequelae Post–Delta or Omicron Variant SARS-CoV-2 Infection in a Highly Vaccinated Cohort. *JAMA Netw Open*. Published August 30, 2024.
doi:10.1001/jamanetworkopen.2024.30983

Data
Data available: Yes
Data types: Other (please specify)
Additional Information: The databases with individual-level information used for this study are not publicly available due to personal data protection. Deidentified data can be made available for research, subject to approval by the Ministry of Health of Singapore. All inquiries should be sent to the corresponding author.
How to access data: Corresponding author email: ian.wee.l.e@singhealth.com.sg
When available: With publication

Supporting Documents
Document types: None

Additional Information
Who can access the data: Deidentified data can be made available for research, subject to approval by the Ministry of Health of Singapore.
Types of analyses: Deidentified data can be made available for research, subject to approval by the Ministry of Health of Singapore.
Mechanisms of data availability: Deidentified data can be made available for research, subject to approval by the Ministry of Health of Singapore.
